# Alpha‐1 Antitrypsin Inclusions Sequester GRP78 in a Bile Acid–Inducible Manner

**DOI:** 10.1111/liv.16207

**Published:** 2024-12-12

**Authors:** Igor Spivak, Nurdan Guldiken, Valentyn Usachov, Frank Schaap, Steven W.M. Olde Damink, Marion Bouchecareilh, Alexandra Lehmann, Lei Fu, Fa‐Rong Mo, Gökce Kobazi Ensari, Franziska Hufnagel, Malin Fromme, Christian Preisinger, Pavel Strnad

**Affiliations:** ^1^ Medical Department III, Gastroenterology, Metabolic Diseases and Intensive Care University Hospital RWTH Aachen Aachen Germany; ^2^ Department of Surgery, Maastricht University Medical Center and NUTRIM School of Nutrition and Translational Research in Metabolism Maastricht University Maastricht Netherlands; ^3^ Department of General, Visceral and Transplant Surgery University Hospital RWTH Aachen Aachen Germany; ^4^ INSERM, CNRS, U1312 BRIC University Bordeaux Bordeaux France; ^5^ Department of Science and Technology Ruikang Hospital Affiliated to Guangxi University of Chinese Medicine Nanning China; ^6^ Interdisciplinary Center for Clinical Research (IZKF) University Hospital RWTH Aachen Aachen Germany

**Keywords:** α‐1 antitrypsin deficiency, cholestatic liver injury, ER chaperone, genetic liver disease, rare liver disease

## Abstract

**Background and Aims:**

The homozygous PiZ mutation (PIZZ genotype) constitutes the predominant cause of severe alpha‐1 antitrypsin (AAT) deficiency and leads to liver disease via hepatocellular AAT aggregation. We systematically analysed the composition of AAT aggregates and studied the impact of bile acids.

**Methods:**

AAT inclusions were isolated from livers of PiZ overexpressing mice and PIZZ humans via fluorescence‐activated and immunomagnetic sorting (FACS/MACS), while insoluble proteins were obtained via Triton‐X extraction. Inclusion composition was evaluated through mass‐spectrometry (MS), immunoblotting and immunostaining. Hepatocytes with versus without AAT aggregates were obtained via microdissection. Serum bile acids were assessed in 57 PIZZ subjects and 19 controls. Mice were administered 2% cholic acid (CA)–supplemented chow for 7 days.

**Results:**

MS identified the key endoplasmic reticulum chaperone 78 kDa glucose‐regulated protein (GRP78) in FACS/MACS pulldowns. GRP78 was also enriched in insoluble fractions from PiZ mice versus wild types and detected in insoluble fractions/MACS isolates from PIZZ liver explants. In cultured cells/primary hepatocytes, PiZ overexpression was associated with increased GRP78 mRNA/protein levels. In human livers, hepatocytes with AAT aggregates had higher GRP78 levels than hepatocytes without. PIZZ subjects displayed higher serum bile acid levels than controls and the highest levels were seen in individuals with liver injury/fibrosis. In PiZ mice, CA‐mediated bile acid challenge resulted in increased liver injury and translocation of GRP78 into the aggregates.

**Conclusions:**

Our results demonstrate that GRP78 is sequestered within AAT inclusions. Bile acid accumulation, as seen in PIZZ subjects with liver disease, may promote GRP78 segregation and thereby augment liver damage.

**Trial Registration:** NCT02929940

**Abbreviations**
AAT: alpha‐1 antitrypsin
AATD: alpha‐1 antitrypsin deficiency
ALT: alanine aminotransferase
AP: alkaline phosphatase
AST: aspartate aminotransferase
Avg: average
BMI: body mass index
CA: cholic acid
Ces3a: carboxylesterase 3a
CHOP: C/EBP homologous protein
Cyt: cytoplasm
EGTA: ethylene glycol bis(2‐aminoethyl)tetraacetic acid
ER: endoplasmic reticulum
FACS: fluorescence‐activated cell sorting
FEV1: forced expiratory volume in one second
FSC: forward scatter
FT: flowthrough
GAPDH: glyceraldehyde‐3‐phosphate dehydrogenase
GGT: gamma‐glutamyltransferase
GRP78: 78 kDa glucose‐regulated protein
GRP94: 94 kDa glucose‐regulated protein
HDAC2: histone deacetylase 2
HRP: horseradish peroxidase
iBAQ: intensity‐based absolute quantification
IEB: isotonic extraction buffer
JNK: c‐Jun N‐terminal kinase
K8: keratin 8
LD: liver disease
Lfib: liver fibrosis
Linj: liver injury
LFQ: label‐free quantification
Lys: lysosome
MACS: magnetic‐activated cell separation
Mem: membrane
Mito: mitochondrial
PAS‐D: periodic acid–Schiff staining with diastase digestion
PVDF: polyvinylidene difluoride
qRT‐PCR: quantitative real‐time polymerase chain reaction
Sec: secreted
SSC: side scatter
TUNEL: terminal deoxynucleotidyl transferase dUTP nick end labelling
ULN: upper limit of normal
UPR: unfolded protein response
WT: wild type

**Summary**

- The homozygous PiZ mutation causes alpha‐1 antitrypsin (AAT) deficiency–associated liver disease due to aggregation of the mutated protein.
- Our data demonstrate that the key endoplasmic reticulum chaperone GRP78 constitutes a major non‐AAT aggregate component.
- Humans with AAT deficiency–related liver injury display elevated serum bile acid levels and when challenged with bile acids, PiZ mice develop an increased liver injury and a translocation of GRP78 into the aggregates.

## 1 Introduction

Protein misfolding and formation of aggregates is a hallmark of multiple neurologic, muscular, hepatic and systemic disorders such as Alzheimer's, Parkinson's or Huntington's disease [1]. The latter ones are the subject of intense research that yielded important insights into the process of aggregate formation [2]. While there is growing evidence that protein aggregation promotes the progression of the corresponding disorder, the exact mechanisms remain incompletely understood [2]. Among the potential underlying causes, aggregates sequester essential proteins and overexpression of the sequestered proteins in a cell‐based model rescued cell viability [3].

Alpha‐1 antitrypsin deficiency (AATD) is an archetypal hepatic proteotoxic disease caused by inherited mutations in the SERPINA1 gene encoding the otherwise secreted protease inhibitor alpha‐1 antitrypsin (AAT). An amino acid substitution (Glu342Lys; rs28929474) in AAT termed PiZ/Z‐AAT protein is responsible for the vast majority of severe AATD cases and leads to an 85% decrease in AAT secretion [4]. Most of the retained AAT is degraded, whereas 15% forms ordered polymers [5] and the corresponding aggregates can be visualised via periodic acid–Schiff staining with diastase digestion (PAS‐D) [6]. The homozygous PiZ mutation (PIZZ genotype) is found in ca. 1:3000 Caucasians and strongly predisposes to early onset lung emphysema as well as liver disease. The latter one displays a biphasic pattern with the first peak in early childhood, where some subjects develop neonatal cholestasis that may progress to end‐stage liver disease [7]. While most of the children survive and become asymptomatic with normalised liver enzyme values in later childhood, PIZZ subjects carry an approximately 20 times increased risk of cirrhosis in later adulthood [8].

The PiZ transgenic mouse model is widely utilised in preclinical studies of AAT aggregation as these mice overexpress human Z‐AAT protein and recapitulate numerous aspects of liver disease associated with human AATD. In the liver, they accumulate the characteristic AAT globules and exhibit chronic liver injury including the development of fibrosis and increased HCC incidence [9].

Although PIZZ subjects are affected by a genetically simple, monogenic disorder, both the liver phenotype and the disease course are highly variable [8]. While the mechanisms leading to the observed biphasic pattern constitute an unresolved AATD mystery, several lines of evidence demonstrate that the PiZ variant confers a susceptibility to cholestatic liver injury and gallstone formation [10, 11]. As a potential underlying mechanism, the hydrophobic bile acid lithocholic acid promotes AAT polymerisation [12], while hydrophilic bile acids decrease intrahepatic AAT content and improve the associated injury [13, 14]. Given that early life is characterised by an immature hepatic excretory function with an increase in serum bile acid levels [15, 16], an imbalance in bile acid repertoire may play a role in neonatal AATD‐associated liver disease. In line with that, treatment of children with AATD‐associated liver disease with ursodeoxycholic acid is widely used although the evidence of its efficacy is limited [17]. Another fact linking protein aggregation with liver disease is the observation that intrahepatic AAT accumulation is higher in subjects with advanced liver fibrosis stages, but the exact relationship between inclusions and liver injury remains unknown [18, 19, 20].

The increasing awareness about AATD, the relatively ease to target hepatocytes and the simple genetic basis led to a surge in clinical trials targeting intrahepatic synthesis and processing of the mutated AAT [21]. Among them, siRNA‐mediated silencing of AAT production results in improvement of liver injury biomarkers in both transgenic animals and humans [20]. In contrast, small molecules modifying hepatocellular AAT folding and processing yielded encouraging data in experimental models but convincing human evidence is lacking [22, 23]. To facilitate the design and clinical testing of these urgently needed therapeutic compounds, a better understanding of the process of protein misfolding, accumulation and its downstream molecular effects is urgently required. As an important step in this direction, a recent study demonstrated that AAT polymers undergo a liquid: solid phase transition, thereby retarding the mobility of endoplasmic reticulum (ER) proteins [24]. To extend these observations, we systematically analysed the composition of AAT aggregates in mice and humans using three complementary methods. To test the potential impact of bile acids, we examined bile acid levels in PIZZ adults and corresponding controls and assessed the impact of CA‐induced cholestatic stress. Our findings demonstrate that 78 kDa glucose‐regulated protein (GRP78), a key ER chaperone, constitutes a major non‐AAT component of AAT inclusions. Moreover, its sequestration increases during bile acid overload that occurs in PIZZ adults with AATD‐associated liver disease.

## 2 Materials and Methods

### 2.1 Human Subjects

Individuals were recruited as a part of our global previously described multicentre alpha‐1 liver registry initiative [25]. Frozen liver specimens were obtained from patients undergoing liver transplantation due to end‐stage liver disease at the University Hospital Aachen (Table S1) while samples from the Tissu‐Tumorothèque Est, CRB‐HCL were used for microdissection experiments [26]. The study was conducted in compliance with the Declaration of Helsinki and Good Clinical Practice guidelines. An approval had been issued by the local ethics review board (EK 173/15) and informed consent was obtained from all subjects prior to the enrolment (also see Data S1).

### 2.2 Mouse Experiments

Transgenic mice on a C57BL/6N background overexpressing the human PiZ‐allele and their nontransgenic littermates (WT) were generated as described [27]. After 2 weeks of acclimatisation, mice were randomised into the different treatment groups. To study bile acid toxicity, the animals were fed either a standard diet or cholic acid–supplemented chow (2% CA in standard diet for 7 days, Ssniff GmbH, Soest, Germany). The experiments were approved by the responsible authority and the applicable institutional and/or national guidelines for the care and use of animals were followed (see Data S1 for details).

### 2.3 Protein Isolation and Analysis

For preparation of total liver lysates, the tissue was homogenised in 3% SDS (Roth, Germany)‐containing buffer supplemented with protease and phosphatase inhibitors (Roche, Mannheim, Germany) and debris was removed by centrifugation. Prior to analysis, the extracts were diluted with 4x reducing Laemmli buffer containing 3% SDS, 125 mM Tris–HCl (pH 6.8), 20% glycerol, 4% β‐mercaptoethanol and 0.01% bromophenol blue.

For isolation of soluble fractions, the liver tissue was immersed in buffer containing 1% Triton‐X and 5 mM EDTA in PBS (Sigma, Munich, Germany) and homogenised using TissueLyser II (Qiagen, Hilden, Germany). After centrifugation at 20000 g for 15 min, the supernatant (soluble fraction) and pellet (insoluble fraction) were diluted with 4x reducing Laemmli buffer. All buffers used during the process were supplemented with protease and phosphatase inhibitors (Roche).

High salt extraction method was performed in human liver explants to obtain insoluble cellular proteins devoid of DNA contamination [28]. Liver tissue was homogenised in buffer containing 1% Triton‐X as described above. The pellet was homogenised with 1.5 mL of high‐salt buffer and incubated for 30 min at 4°C on a shaker. The pellets were washed twice in 1xPBS‐EDTA solution and collected at 20000 g for 30 min. Finally, pellets were dissolved in 4x reducing Laemmli buffer and protein extracts were separated via SDS‐PAGE.

For subcellular fractionation, the liver tissue was dispersed in isotonic extraction buffer (IEB) containing 10 mM HEPES, 250 mM sucrose, 25 mM potassium chloride and 1 mM ethylene glycol bis(2‐aminoethyl)tetraacetic acid (EGTA, Sigma). The samples were homogenised and centrifuged twice at 2000 g for 5 min to obtain a pure nonnuclear fraction in the supernatant and a pure nuclear fraction in the pellet. Both fractions were dissolved in 4x reducing Laemmli buffer.

Proteins were separated by 10% SDS‐PAGE. Coomassie blue staining was performed according to the manufacturer's instructions (Thermo Fischer). For immunoblotting, proteins were transferred to polyvinylidene difluoride (PVDF) membranes (GE Healthcare, Solingen, Germany). Membranes were blocked and incubated with primary and horseradish peroxidase (HRP)‐conjugated secondary antibodies listed in Table S2. The HRP signal was visualised by enhanced chemiluminescence (GE) or ChemiDoc imager system with the Image Lab software as recommended by the manufacturer (Bio‐Rad Laboratories, Hercules, CA, USA). Image quantification was performed with ImageJ (NIH, Bethesda, MD, USA).

### 2.4 Cell Culture Experiments

Primary hepatocytes were isolated as described previously [29] from 10‐ to 12‐week‐old male PiZ and WT mice. Cells from a human bronchial epithelial line (IB3) were transfected with either wild‐type (WT) or Z‐variant AAT [26] (see Data S1 for details).

### 2.5 Statistical Analysis

Researchers were blinded to the group allocation during data analysis. All animals involved in the experimental procedures were included in the analyses and no data points collected during the study were excluded. For comparisons between two groups, unpaired two‐tailed Student's *t*‐test or unpaired Mann–Whitney *U* test was used depending on the distribution. Results were described as mean ± SD or median ± IQR. For multiple comparisons, ordinary one‐way analysis of variance with Fisher's least significant difference or Kruskal–Wallis with Dunn's test was used depending on the distribution. Multivariate hypothesis testing for bile acid composition data was done using permutational analysis of variance (PERMANOVA). False discovery rate adjustment was performed using the Benjamini–Krieger–Yekutieli procedure. Statistical significance was defined as a two‐sided *p*‐value < 0.05. Statistical analyses were performed with Prism version 9.5.1 (GraphPad, LaJolla, CA, USA) or R with Metaboanalyst package.

Further experimental procedures (e.g., proteomics, fluorescence‐activated cell sorting (FACS), magnetic‐activated cell sorting (MACS), quantitative real‐time polymerase chain reaction (qRT‐PCR), histology and bile acid measurement) are given in the Data S1.

## 3 Results

### 3.1 Purification of Aggregated Proteins

**FIGURE 1:**
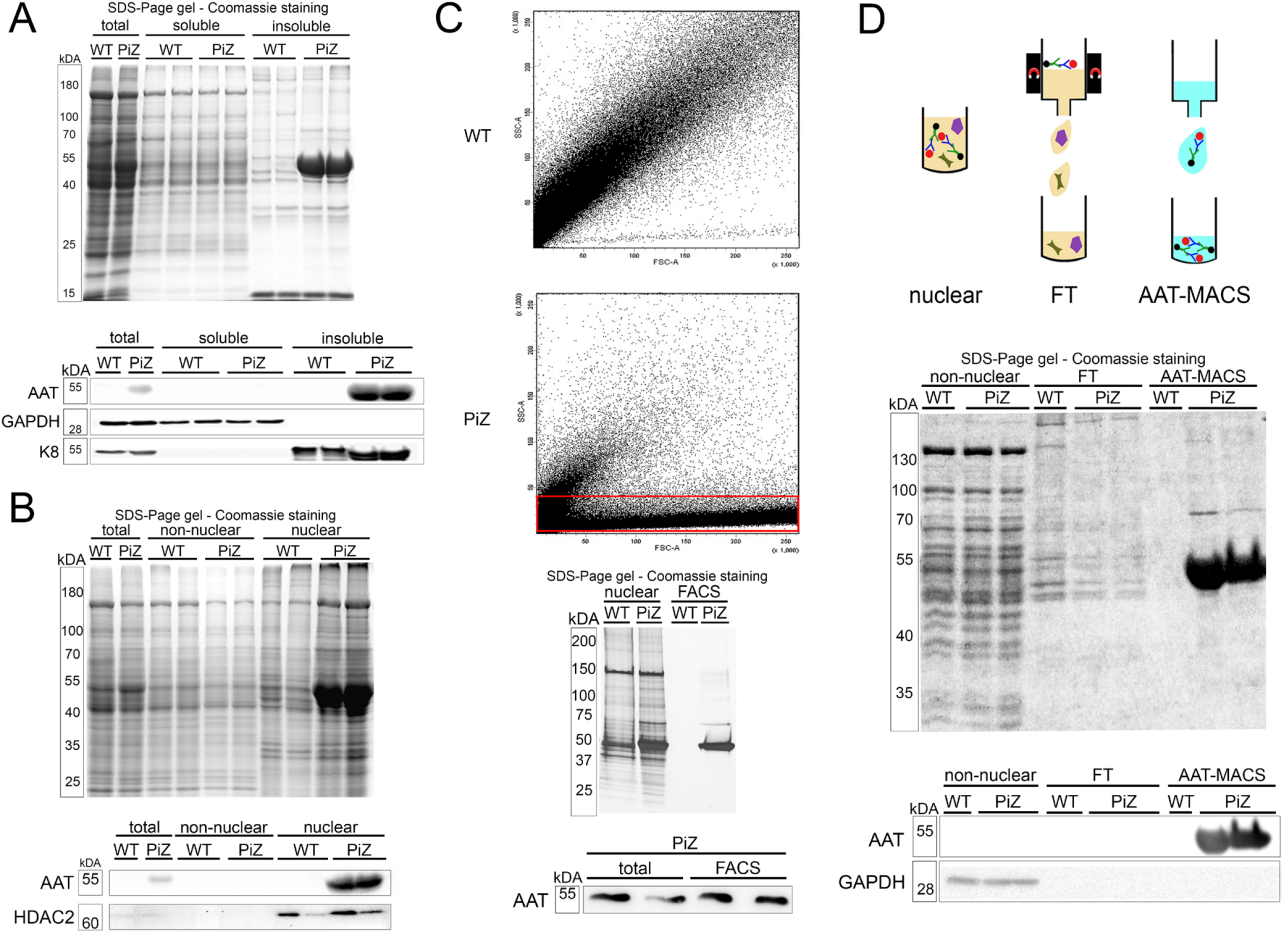
Purification of aggregated proteins from PiZ mouse livers. (A) Total liver lysates, Triton‐X soluble and insoluble fractions from PiZ transgenic and nontransgenic littermates (WT) were separated by SDS‐PAGE and stained with Coomassie blue. Immunoblot analysis was performed with an antibody against AAT. GAPDH and K8 were used as controls for loading and fractionation. (B) Fractionation of proteins from PiZ and WT livers, followed by separation via SDS‐PAGE and Coomassie‐staining or immunoblotting with an antibody against AAT and the nuclear protein HDAC2. (C) The nuclear isolates underwent FACS. A unique fraction of particles seen in PiZ but not WT samples is highlighted with a rectangle. SDS‐PAGE and Coomassie staining was performed to visualise the protein composition, while immunoblotting quantified the AAT content. (D) Nuclear fractions from PiZ and WT mouse livers underwent labelling with AAT antibody and a pulldown with secondary antibodies‐coupled magnetic beads (AAT‐MACS). The flowthrough (FT) contained the unbound proteins. The different fractions underwent SDS‐PAGE separation, Coomassie staining as well as immunoblotting with an antibody against AAT and the cytoplasmic protein GAPDH. AAT, alpha‐1 antitrypsin; FACS, fluorescence‐activated cell sorting; FSC, forward scatter; FT, flowthrough; GAPDH, glyceraldehyde‐3‐phosphate dehydrogenase; HDAC2, histone deacetylase 2; K8, keratin 8; MACS, magnetic activated cell separation; SSC, side scatter.

To determine the composition of AAT aggregates, we studied 2‐ to 3‐month‐old PiZ mice and their nontransgenic (WT) littermates. Compared to WT animals, PiZ mice had significantly higher alanine aminotransferase (ALT) levels (39 ± 21 vs. 27 ± 12 U/L; *p* < 0.01; Figure S1), but no difference in alkaline phosphatase (AP) and aspartate aminotransferase (AST) levels (Figure S1). Histologic analyses revealed abundant PAS‐D‐positive inclusions in PiZ mice, but a largely preserved liver architecture and no signs of fibrosis (Figure S1). Given that Z‐AAT is largely insoluble in nonionic detergents, we performed a fractionation of liver proteins into Triton X‐soluble and ‐insoluble pools. Coomassie staining and immunoblotting confirmed the presence of AAT in the insoluble fraction of PiZ but not in WT livers (Figure 1A). For direct isolation of aggregates, we first enriched Z‐AAT inclusions via subcellular fractionation. Coomassie staining and immunoblotting demonstrated that AAT is present almost exclusively in the nuclear fraction of PiZ mice (Figure 1B). PAS‐D‐staining confirmed abundant inclusions in the nuclear fraction from PiZ mice, while no aggregates were seen in the fractions derived from WT animals, nor in the nonnuclear compartment from PiZ mice (Figure S2). Subsequent fluorescence‐activated cell sorting (FACS) of nuclear isolates from PiZ mice revealed a distinct fraction of particles with low side scatter (SSC) signal and a broad range of forward scatter (FSC) intensities which was not present in the isolates from WT mouse livers (Figure 1C). Collection of this fraction with subsequent Coomassie staining and immunoblotting revealed a largely pure AAT fraction in PiZ mice and almost no proteins in WT animals (Figure 1C). Finally, we applied MACS as a complementary method for the isolation of AAT aggregates. To that end, inclusions in the nuclear fraction were labelled with AAT antibody and pulled down with magnetic nanoparticles coupled with corresponding secondary antibodies (Figure 1D). Coomassie staining of the isolates revealed a picture reminiscent of the one seen after FACS isolation (Figure 1D). The presence of AAT in fractions from PiZ mice was confirmed by immunoblotting (Figure 1D). Collectively, three independent methods allowed us to isolate Z‐AAT‐enriched fractions as a tool to study the aggregate composition.

### 3.2 GRP78 Represents a Major Component of AAT Inclusions

**FIGURE 2:**
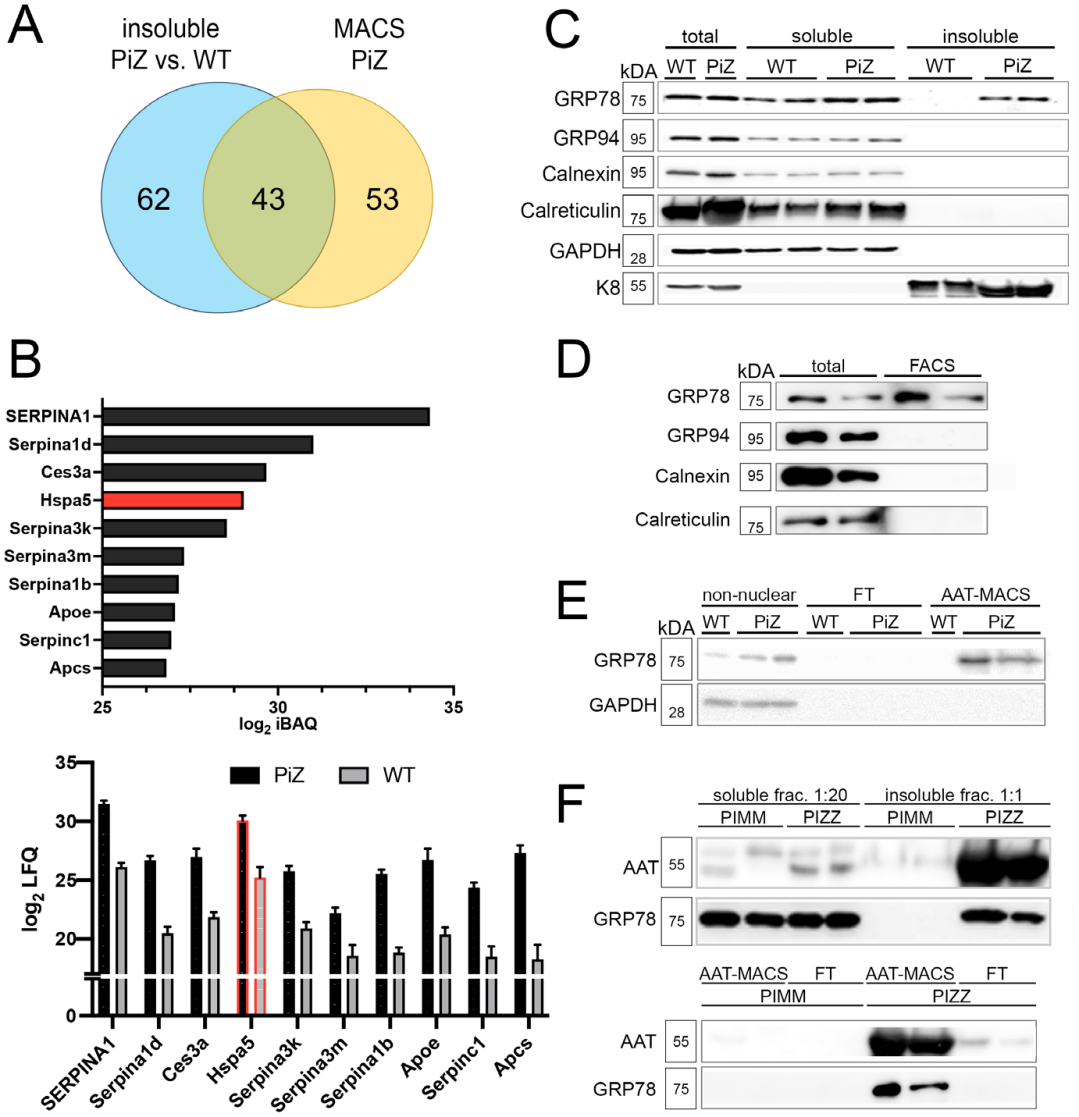
Identification of GRP78 as a component of mouse and human AAT aggregates. (A) Venn diagram illustrating the number of proteins identified by mass spectrometry to be enriched in the insoluble fractions of livers from PiZ transgenic mice versus nontransgenic littermates (WT) (blue circle) and present in the magnetic beads–mediated pulldown of AAT‐containing particles (MACS) from PiZ mouse livers (yellow circle). Out of the overlapping proteins, (B) illustrates the log_2_ intensity‐based absolute quantification (iBAQ) values of the proteins most abundant in the MACS pulldowns from PiZ mouse livers (upper panel) and shows log_2_ label‐free quantification (LFQ) values of these proteins in insoluble fractions from PiZ and WT mouse livers (lower panel). (C–E) Immunoblot analysis was performed with antibodies against GRP78 and other endoplasmic reticulum chaperones in the insoluble and soluble pool of PiZ and WT mouse livers (C), FACS isolates from nuclear fractions of PiZ mouse livers (D) and MACS pulldowns from nuclear fractions of PiZ and WT mouse livers (E). (F) Human liver explants from PIZZ individuals and subjects without the PiZ variant (PIMM genotype) were subdivided into insoluble and soluble fractions (upper panel) or subjected to MACS pulldown of nuclear fractions (lower panel) followed by immunoblotting with antibodies against AAT and GRP78. GAPDH and K8 were used as controls for loading and fractionation. AAT, alpha‐1 antitrypsin; FACS, fluorescence‐activated cell sorting; GAPDH, glyceraldehyde‐3‐phosphate dehydrogenase; GRP78, 78 kDa glucose‐regulated protein; GRP94, 94 kDa glucose‐regulated protein; iBAQ, intensity‐based absolute quantification; K8, keratin 8; LFQ, label‐free quantification; MACS, magnetic‐activated cell separation.

To identify proteins trapped in Z‐AAT aggregates, we examined the Triton X‐insoluble fractions and MACS pulldowns from PiZ and WT mice by mass spectrometry. A total of 105 proteins were enriched in the insoluble fractions from PiZ versus WT mice, whereas 96 proteins were identified in MACS isolates from PiZ mouse livers (Figure 2A) and both analyses yielded 43 shared proteins (Table 1). Apart from AAT and homologous Serpina proteins, the most abundant hits were carboxylesterase 3a (Ces3a) and 78 kDa glucose‐regulated protein (GRP78) (also known as BiP or HSPA5) (Figure 2B). While Ces3a is a relatively poorly studied ER enzyme hydrolysing lipid esters and contributing to lipoprotein synthesis in the liver [30], GRP78 is a key ER chaperone responsible for the handling and fate of misfolded proteins, including AAT [31, 32]. Immunoblotting confirmed the accumulation of GRP78 in the Triton X‐insoluble fraction of PiZ but not WT mouse livers (Figure 2C). We did not observe a compensatory decrease of the GRP78 signal in the soluble fraction of PiZ mouse livers. As most of the cellular GRP78 is found in the soluble fraction, changes seen in the insoluble fraction are likely under the detection sensitivity of the immunoblotting. Notably, none of the other analysed ER chaperones including calnexin, calreticulin or 94 kDa glucose‐regulated protein (GRP94) were detected in the insoluble fraction (Figure 2C). An accumulation of GRP78 was also seen in insoluble fractions from cultured primary hepatocytes from PiZ mice that displayed abundant PAS‐D‐positive inclusions (Figure S3). Similar to the insoluble fractions, immunoblotting of FACS isolates revealed GRP78 but none of the other assessed ER chaperones to be enriched in extracts from PiZ versus WT mouse livers (Figure 2D). A similar finding was obtained when MACS was used as an alternative pulldown method (Figure 2E). When insoluble fractions and MACS pulldowns from PiZ mouse livers were compared side‐by‐side, a similar AAT‐GRP78 stoichiometry was seen (Figure S4). To test, whether the experimental findings translate to humans, we assessed individuals without (PIMM) and with homozygous PiZ mutation (PIZZ). Explants from PIZZ subjects with end‐stage liver disease (Table S1) displayed a strong AAT and a clearly detectable GRP78 signal in the insoluble hepatic fraction, while both proteins were found only in the soluble hepatic pool in adult PIMM individuals with end‐stage liver disease (Figure 2F). Since PIZZ genotype can lead to severe liver disease in early childhood, we assessed GRP78 in a paediatric PIZZ subject and demonstrated the sequestration of GRP78 in the insoluble pool (Figure S5). A MACS‐based pulldown of AAT inclusions confirmed the presence of GRP78 in isolates from individuals with PIZZ but not PIMM genotype (Figure 2F). Thus, several independent methods demonstrated that the ER‐chaperone GRP78 represents a major component of AAT inclusions both in humans and mice.

**TABLE 1:** Proteins enriched in both insoluble fractions and magnetic‐activated cell separation (MACS) isolates from PiZ mouse livers.

			AAT‐MACS	Insoluble fraction				
Gene name	Majority protein IDs	Subcellular localisation	log_2_ avg iBAQ PiZ	Avg LFQ intens. PiZ	Avg LFQ intens. WT	Ratio	−log_10_ *t*‐test *p*	log_2_ avg iBAQ PiZ
SERPINA1	P01009	ER	34.32	31.50	26.13	41.29	10.55	32.52
Serpina1d	Q00897	Sec	31.01	26.69	20.49	73.69	9.30	26.69
Ces3a	Q63880	ER	29.67	26.98	21.85	35.00	7.56	28.22
Hspa5	P20029	ER	29.03	30.09	25.22	29.29	6.59	27.47
Serpina3k	P07759	Sec	28.54	25.76	20.87	29.50	7.92	26.24
Serpina3m	Q03734	Sec	27.33	22.21	18.56	12.51	5.28	22.20
Serpina1b	P22599	Sec	27.18	25.53	18.84	103.75	10.36	24.96
Apoe	P08226	Sec	27.07	26.72	20.39	80.67	7.14	26.13
Serpinc1	P32261	Sec	26.96	24.35	18.49	58.30	7.34	24.04
Apcs	P12246	Sec	26.83	27.33	18.27	533.56	7.74	26.09
Serpina1e	Q00898	Sec	25.94	25.03	15.80	599.24	10.13	25.31
Serpina3n	Q91WP6	Sec	25.10	22.31	18.59	13.14	6.38	22.34
Fgg	Q8VCM7	Sec	23.82	23.84	22.75	2.13	1.33	22.96
Alb	P07724	Sec	23.70	24.75	23.13	3.08	3.04	22.17
Inmt	P40936	Cyt	23.59	23.87	19.78	16.98	3.59	22.48
Serpinf1	P97298	Sec	23.32	22.60	18.74	14.54	5.15	21.70
Ctsd	P18242	Lys	22.86	21.64	19.27	5.18	2.27	19.99
Mbl2	P41317	Sec	22.86	22.96	18.26	26.03	7.63	22.07
Entpd5	Q9WUZ9	ER	22.74	23.73	19.13	24.31	7.54	22.09
Clu	Q06890	Sec	22.67	23.01	19.87	8.80	4.22	20.99
Mup2	P11589	Sec	22.57	26.34	19.91	85.77	4.45	23.93
Apoa1	Q00623	Sec	22.55	25.05	20.30	26.86	3.86	23.73
Mbl1	P39039	Sec	22.53	22.89	18.20	25.91	6.53	21.95
Crp	P14847	Sec	22.53	23.17	18.12	33.01	6.69	22.28
Tf	Q921I1	Sec	22.53	23.44	16.67	109.80	5.06	22.31
Rbp4	Q00724	Sec	22.32	26.11	17.96	283.09	10.81	24.75
Serpind1	P49182	Sec	22.12	24.33	18.26	67.17	7.31	21.86
Gnmt	Q9QXF8	Cyt	21.96	22.56	20.77	3.47	5.53	20.67
Ces1c	P23953	ER	21.76	21.82	18.15	12.74	5.69	20.22
Azgp1	Q64726	Sec	21.55	22.80	18.33	22.15	6.90	21.65
Sil1	Q9EPK6	ER	21.48	20.64	19.15	2.80	2.38	19.97
Plg	P20918	Sec	21.16	23.66	20.88	6.84	4.54	21.03
C3	P01027	Sec	21.10	23.23	18.65	23.86	5.34	20.23
Dnajb9	Q9QYI6	ER	20.94	23.46	18.83	24.83	6.02	22.08
Ctsl	P06797	Lys	20.93	21.43	18.29	8.78	5.43	20.22
Serping1	P97290	Sec	20.64	23.14	18.22	30.29	7.66	19.59
Gc	P21614	Sec	20.56	22.82	18.39	21.58	6.06	20.98
Hs6st1	Q9QYK5	Mem	20.14	24.53	19.15	41.48	7.60	20.33
Fgl1	Q71KU9	Sec	19.73	20.86	18.56	4.91	4.19	19.20
Mug2	P28666	Sec	19.56	22.46	18.08	20.93	5.12	19.25
Ldha	P06151	Cyt	19.48	21.65	20.40	2.38	3.05	19.16
Hyou1	Q9JKR6	ER	18.73	21.92	19.55	5.15	2.55	16.91
Mug1	P28665	Sec	16.52	20.27	18.44	3.56	3.36	14.91

Abbreviations: AAT, alpha‐1 antitrypsin; Avg, average; Cyt, cytoplasm; ER, endoplasmic reticulum; iBAQ, intensity‐based absolute quantification; LFQ, label‐free quantification; Lys, lysosome; Mem, membrane; Mito, mitochondrial; Sec, secreted.

### 3.3 Z‐AAT Retention is Associated With Upregulation of GRP78

**FIGURE 3:**
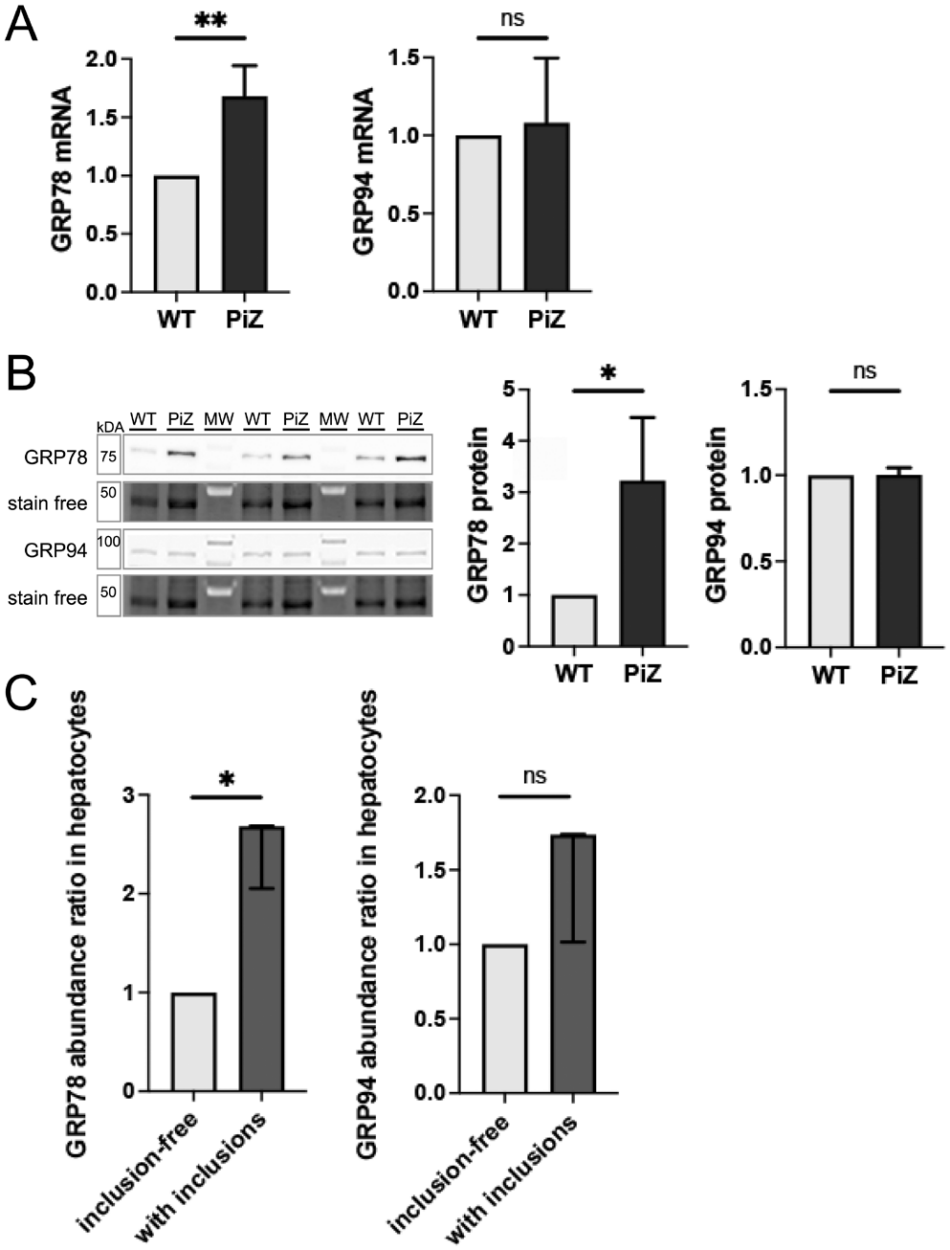
GRP78 and GRP94 levels in a human‐derived bronchial epithelial cell line (IB3) expressing wild‐type (WT) alpha‐1 antitrypsin (AAT) or its PiZ variant and in microdissected human PIZZ liver specimen. (A) GRP78 (*n* = 4 per group) and GRP94 mRNA (*n* = 3 per group) levels were quantified by RT‐qPCR in WT‐ and PiZ‐IB3 cells, 18s ribosomal RNA was used as an internal reference. (B) Immunoblotting against GRP78 and GRP94 (*n* = 3 per group), with corresponding stain‐free gels for normalisation, was used for band intensity quantification. (C) A human liver explant from a subject with PIZZ genotype was PAS‐D stained and laser microdissection was carried out to collect hepatocytes with and without Z‐AAT inclusions. GRP78 and GRP94 abundance ratios were quantified with mass‐spectrometry analysis. Levels of GRP78 and GRP94 in controls were arbitrarily set as 1 and levels in Z‐AAT groups expressed as ratio. Results are shown as mean ± SD (A–B) or median ± range (C). *p < 0.05; **p < 0.01. AAT, alpha‐1 antitrypsin; GRP78, 78 kDa glucose‐regulated protein; GRP94, 94 kDa glucose‐regulated protein; MW, molecular weight marker lane.

Trapping of GRP78 in misfolded protein complexes has been shown to induce an increase of its expression [33] and may also lead to unfolded protein response [34]. To study this further, we assessed the direct effect of PiZ in a human cell line (IB3) overexpressing Z‐AAT. An increase in both GRP78 mRNA and protein levels was detected in cells overexpressing Z‐AAT versus WT AAT (Figure 3A,B). Notably, levels of GRP94, an ER‐chaperone involved in Z‐AAT degradation [34], were not affected (Figure 3A,B). These data suggest that GRP78 induction constitutes a conserved response to Z‐AAT. To test this further, hepatocytes with versus without AAT aggregates were obtained from a PIZZ patient and examined via mass spectrometry. An enrichment of GRP78, but only a trend towards GRP94 increase that did not reach statistical significance was seen in inclusion‐bearing compared to inclusion‐free hepatocytes (Figure 3C). Collectively, this demonstrates that GRP78 levels selectively increase in response to Z‐AAT expression/accumulation.

### 3.4 Bile Acids and Z‐AAT‐Associated Liver Disease

**FIGURE 4:**
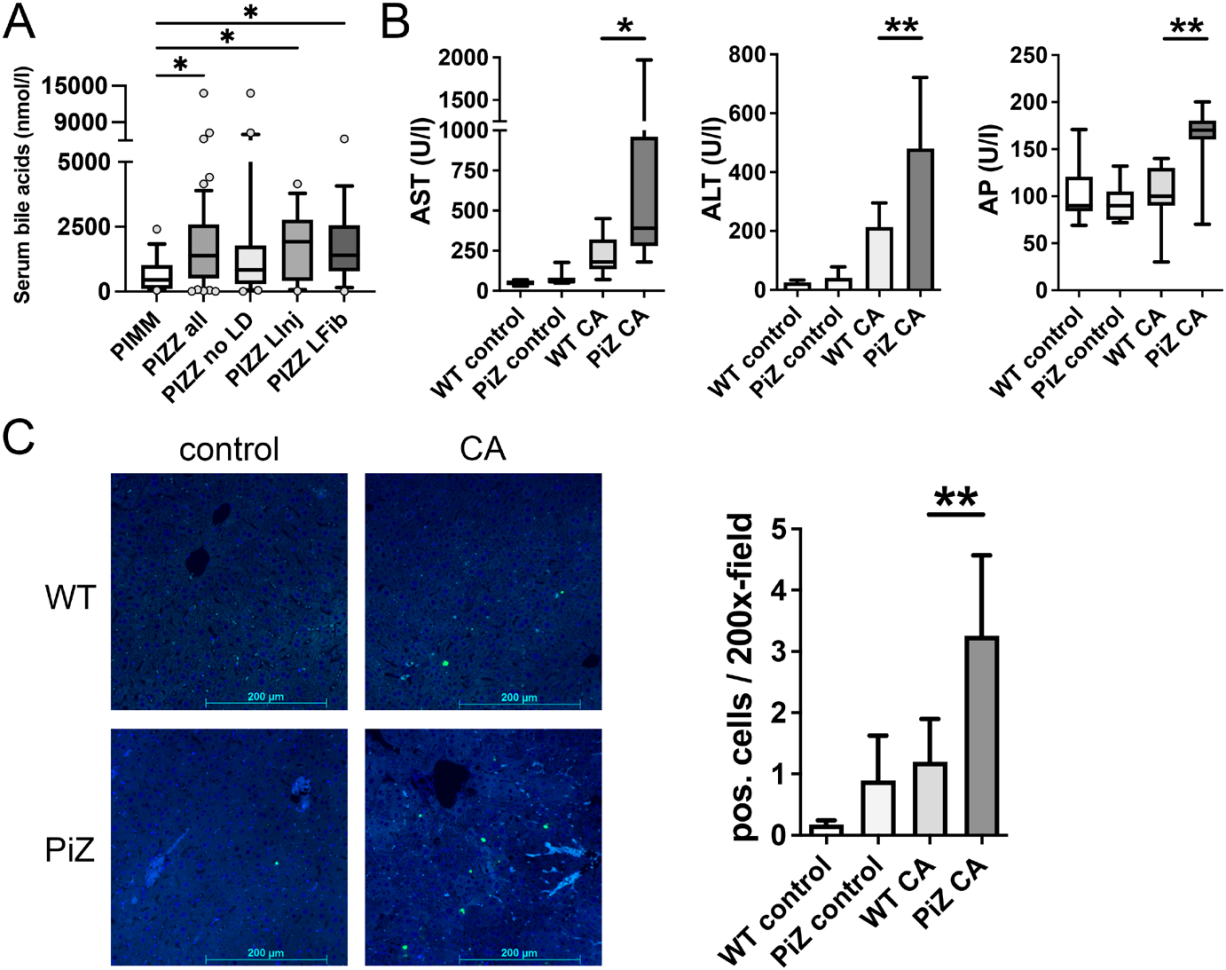
Bile acids in PIZZ adults and cholic acid challenge in PiZ mice. (A) Total serum bile acid concentrations in subjects without the PiZ variant (PIMM genotype) and PIZZ individuals without liver disease (no LD), with elevated liver enzymes but no fibrosis (LInj) or with significant liver fibrosis (LFib). Boxes display median ± IQR and whiskers indicate the 10–90 percentile. Outliers are depicted by circles. (B) Serum AST, ALT and AP levels were measured in PiZ transgenic mice and nontransgenic littermates (WT) fed with normal (control) or 2% cholic acid (CA)–supplemented chow. TUNEL‐staining of liver sections (C) with corresponding quantification. Scale bar = 200 μm. AST and AP are displayed with boxes indicating median ± IQR and whiskers showing the range of the data, ALT and TUNEL‐positive cells as mean ± SD. *p < 0.05; **p < 0.01. ALT, alanine aminotransferase; AP, alkaline phosphatase; AST, aspartate aminotransferase, CA, cholic acid; TUNEL, terminal deoxynucleotidyl transferase dUTP nick end labelling.

**FIGURE 5:**
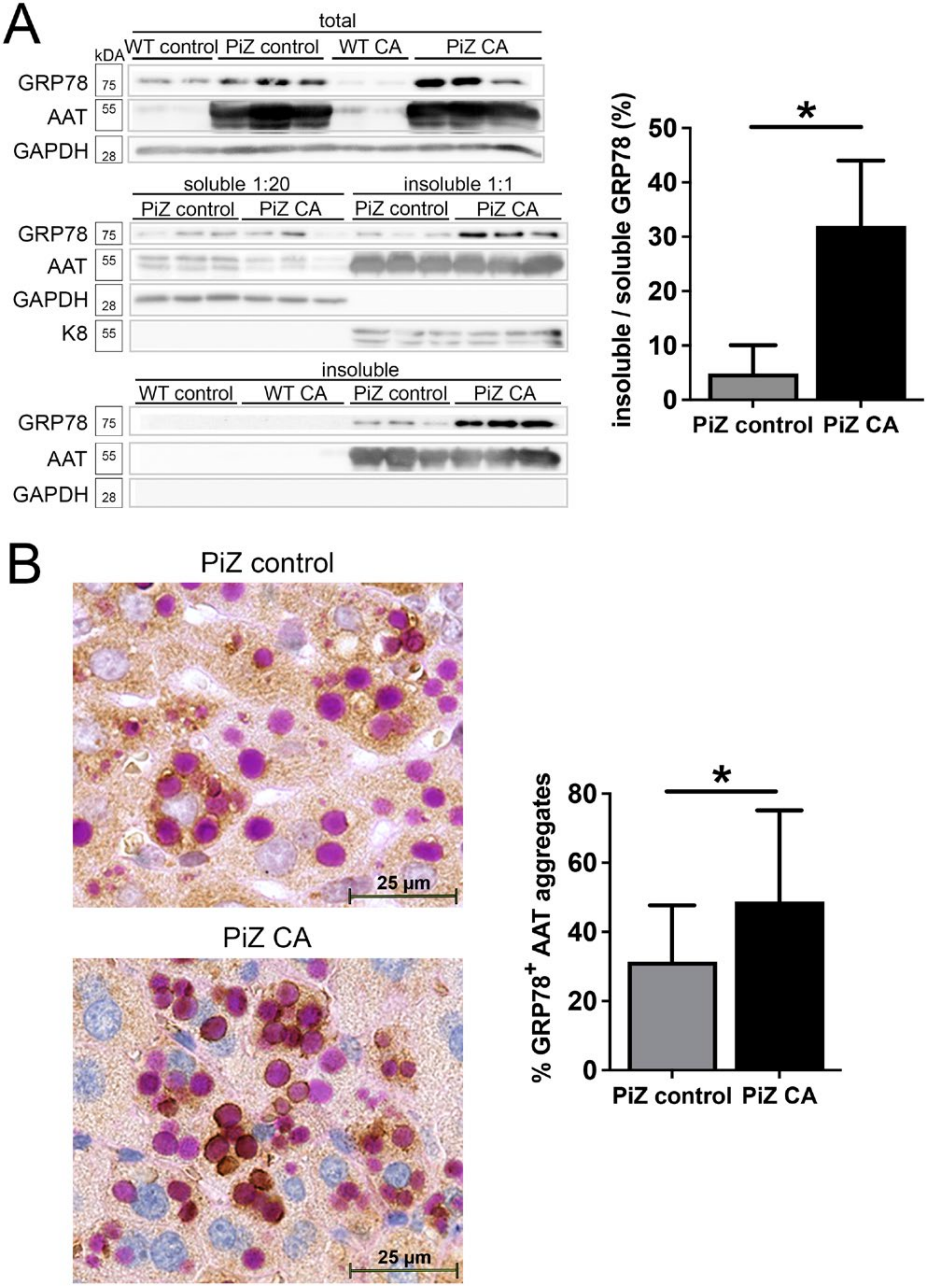
Effect of bile acid challenge on composition of AAT aggregates. (A) Immunoblotting against AAT and GRP78 was performed in the total liver lysates, soluble and insoluble fractions from untreated PiZ transgenic mice (control) and transgenic animals fed with 2% cholic acid (CA)–supplemented chow. GAPDH and K8 were used as controls for loading and fractionation. Band intensity quantification demonstrates the ratio of GRP78 in the insoluble versus soluble pool of PiZ mice. (B) PAS‐D staining combined with GRP78 immunohistochemistry was used to demonstrate colocalisation of GRP78 with the AAT aggregates in PiZ control and CA‐fed animals. Scale bar = 25 μm. Quantification of total AAT aggregates and those with positive GRP78 staining shows the ratio of GRP78^+^ aggregates in PiZ mice. Results are displayed as mean ± SD. *p < 0.05. AAT, alpha‐1 antitrypsin; CA, cholic acid; GAPDH, glyceraldehyde‐3‐phosphate dehydrogenase; GRP78, 78 kDa glucose‐regulated protein; K8, keratin 8; PAS‐D, periodic acid–Schiff‐diastase.

Since AATD‐associated liver disease constitutes an established cholestatic condition in children and accumulation of bile acids was previously shown to promote AAT polymerisation [10, 12, 17], we analysed serum bile acids in 19 PIMM and 57 PIZZ adults (Table 2). Compared to PIMM individuals, PIZZ subjects showed significantly increased total bile acid serum levels (Figure 4A). Absolute values were highest in PIZZ subjects with elevated liver enzyme levels as well as pre‐existing fibrosis determined via a liver stiffness measurement [25] (Figure 4A). In contrast, bile acid levels in those with no liver disease were comparable to the PIMM controls (Figure 4A). We also profiled serum bile acids in PIZZ and PIMM individuals, with PCA demonstrating a significant difference between PIZZ and PIMM subjects (PERMANOVA *F*‐value 9.95, *R* ^2^ 0.11, *p* = 0.001; Figure S6). We identified four bile acids that were significantly enriched in PIZZ subjects: taurochenodeoxycholic acid, taurocholic acid, glycoursodeoxycholic acid and taurodeoxycholic acid (Figure S6). Given the observed association between AATD‐associated liver injury and bile acid levels, we studied whether a bile acid challenge promotes Z‐AAT‐associated liver injury and alters aggregate composition. Feeding with 2% CA‐containing diet over 7 days resulted in significantly higher levels of AST (390 ± 680 vs. 180 ± 185 U/L; *p* < 0.05), moderately, yet significantly elevated ALT (480 ± 241 vs. 213 ± 82 U/L, *p* < 0.01) and AP (170 ± 20 vs. 100 ± 43 U/L; *p* < 0.01) in PiZ mice compared to WT littermates (Figure 4B) despite the fact that bile acid levels in serum and liver were comparable in both CA‐treated groups (Figure S7). Among CA‐treated mice, analysis of terminal deoxynucleotidyl transferase dUTP nick end labelling (TUNEL)‐stained liver sections demonstrated higher rates in PiZ versus WT mice (3.3 ± 0.5 vs. 1.2 ± 0.3 pos. cells/200x‐field; *p* < 0.01; Figure 4C). No significant differences were found among untreated groups (0.9 ± 0.7 vs. 0.2 ± 0.1 pos. cells/200x‐field; *p* = 0.17; Figure 4C). In transgenic animals, CA treatment decreased both AAT mRNA and serum levels (Figure S8), while AAT protein levels as well as the amount of insoluble AAT remained unaltered (Figure 5A). Treated and untreated PiZ mice showed similar total GRP78 levels (Figure 5A), while the ratio of insoluble to soluble GRP78 increased significantly after CA treatment (4.8 ± 5.2 vs. 32% ± 12%; *p* < 0.05, Figure 5A). In WT mice, GRP78 remained undetectable in the insoluble pool (Figure 5A). A combination of PAS‐D staining and GRP78‐immunostaining confirmed the findings in that it revealed a stronger colocalisation of both signals in CA‐treated versus untreated PiZ mice (48.8 ± 26.4 vs. 31.4% ± 16.3%; *p* < 0.05, Figure 5B). We also costained liver sections from an adult and a paediatric PIZZ subject with PAS‐D and GRP78 immunohistochemistry and demonstrated a colocalisation of both signals (Figure S9).

**TABLE 2:** Characteristics of analysed subjects without (PIMM genotype) and with homozygous PiZ variant (PIZZ genotype) in alpha‐1 antitrypsin gene.

Characteristics	PIMM (*n* = 19)	PIZZ (all; *n* = 57)	PIZZ no LD (*n* = 20)	PIZZ LInj (*n* = 19)	PIZZ LFib (*n* = 18)
Age, mean (SD), years	45.8 (14.4)	46.7 (14.2)	44.9 (11.5)	45.9 (16.6)	49.4 (14.6)
Woman, *n* (%)	7 (35)	20 (35)	7 (35)	7 (37)	6 (33)
BMI, mean (SD), kg/m^2^	24.4 (2.8)	24.5 (3.6)	24.3 (3.7)	24.0 (3.2)	25.3 (4.0)
AAT serum level, median (IQR), mg/dl	117.5 (19.8)	23.1 (11.3)***	20.8 (3.9)***	26.9 (19.1)**	25.6 (15.2)*
Liver stiffness, mean (SD), kPa	4.4 (1.0)	6.5 (2.5)***	5.1 (0.8)	5.1 (1.0)	9.6 (2.2)***
AST median (IQR), % of ULN	54.0 (16.5)	77.1 (36.1)***	57.0 (18.1)	102.9 (34.0)***	82.0 (30.6)***
ALT median (IQR), % of ULN	53.0 (27.6)	88.6 (57.5)***	52.0 (27.4)	114.0 (67.4)***	81.4 (54.6)**
GGT median (IQR), % of ULN	40.0 (20.6)	58.3 (43.3)***	53.3 (28.8)	63.3 (65.0)***	77.9 (103.1)***
AP median (IQR), % of ULN	53.2 (19.7)	57.7 (25.4)	55.0 (28.7)	57.7 (18.1)	62.4 (33.1)
Bilirubin median (IQR), mg/dl	0.49 (0.33)	0.60 (0.29)	0.46 (0.34)	0.61 (0.43)	0.66 (0.21)

*Note:* PIZZ no LD—no liver disease (liver stiffness < 7.1 kPa, unaltered liver enzymes). PIZZ LInj—no significant liver fibrosis, but elevated liver enzymes (at least ALT≥ ULN, liver stiffness < 7.1 kPa). PIZZ LFib—significant liver fibrosis but no decompensated cirrhosis (liver stiffness ≥ 7.1 kPa). Only subjects without an existing lung injury were examined (forced expiratory volume in one second [FEV1] > 70%). Normally distributed values are shown as mean (SD), others as mean (IQR). Differences to PiMM group are highlighted.

Abbreviations: AAT, alpha‐1 antitrypsin; ALT, alanine aminotransferase; AP, alkaline phosphatase; AST, aspartate aminotransferase; BMI, body mass index; GGT, gamma‐glutamyltransferase; ULN, upper limit of normal.

* *p* < 0.01.

** *p* < 0.001.

*** *p* < 0.0001.

In conclusion, our data demonstrate that liver injury/fibrosis is associated with increased serum bile acid levels and an altered bile acid composition in PIZZ adults and that bile acid challenge can promote liver injury as well as translocation of the ER‐chaperone GRP78 into the Z‐AAT aggregates.

## 4 Discussion

AATD is an increasingly recognised cause of chronic liver disease [4, 8, 35] and while there is a clear link between AAT accumulation and liver injury [24, 36], the underlying disease mechanisms remain incompletely understood. To change that, we used several methods to isolate AAT inclusions from liver tissues. We took advantage of the insolubility of PiZ in nonionic detergents as well as a comparably large size of AAT inclusions that have been used for isolation for various other protein aggregates [37, 38]. By using this technique, we identified GRP78 as a major constituent of AAT globules. In contrast to our analyses, previous studies were mostly conducted in PiZ overexpressing cell lines and the results regarding GRP78 were inconclusive [24, 39]. While GRP78 constituted a major non‐AAT component of the aggregates, the inclusions did not contain calnexin/calreticulin that are established components of the folding machinery [40]. The presence of GRP78 and absence of calnexin/calreticulin suggest that the isolated particles were properly off‐loaded from the secretory pathway and entered the degradation route [35]. Notably, we did not detect comparable alterations in 2525 GRP94, another ER chaperone implicated in degradation of mutated AAT [34], nor components of degradation machineries. While the importance of autophagy for degradation of AATD inclusions is clearly established [41], autophagic components do not directly interact with misfolded AATD and may, therefore, get lost during the isolation process. On the other hand, ER‐associated degradation, another well‐known AAT disposal pathway [42], is primarily involved in destruction of early AATD assemblies that were not targeted by our approach [36]. While further studies are needed to address the relevance of early stages of protein aggregation, our approach enabled us to collect > 95% of retained AAT and is, therefore, useful to uncover proteins trapped in AATD inclusions, which was the primary goal of our analyses.

At the basal level, the stoichiometry of sequestered GRP78 was well below 10% of total mouse protein abundance and did not affect the total levels of cellular GRP78, which is in line with literature [43]. This is not surprising since visible AAT aggregates are found only in a minority of hepatocytes and the stoichiometry of retained GRP78 in these cells is likely much higher. Moreover, a depletion of GRP78 is known to initiate an unfolded protein response (UPR) and is embryolethal in transgenic mice [44]. Obviously, this does not occur in PIZZ subjects since adult AATD‐associated liver disease develops only in a subset of PIZZ individuals and constitutes a slowly progressive disorder with normal or mildly elevated liver enzyme levels [8]. However, the situation is different in a small fraction of children who display a rapid disease course and require liver transplantation early in life [17] and in the corresponding mouse model with high AAT load [45]. In the widely used PiZ mice that serve as a model of adult AATD, an obvious UPR is not seen under basal conditions, but may occur after a second hit [46]. Therefore, it is tempting to speculate that a critical GRP78 depletion may take place in a small subset of cells with massive AAT accumulation and/or under specific stress situations. To support the relevance of GRP78 for human pathology, heterozygous mice with liver‐specific GRP78 depletion developed liver steatosis and apoptosis and are susceptible to a variety of liver insults [44]. This is reminiscent of AATD, since PIZZ individuals display increased liver steatosis and apoptosis and both processes are implicated in the disease development in PiZ mice [25, 35]. Notably, the magnitude of apoptosis correlates with the absolute amount of mutated protein found in the individual hepatocyte [47]. Therefore, a high AAT load may deplete cellular GRP78 levels with a resulting UPR and initiation of apoptosis. In that respect, c‐Jun N‐terminal kinase (JNK) signalling pathway and C/EBP homologous protein (CHOP) overexpression are well‐established downstream effectors of UPR with a proven involvement in AATD‐associated liver injury [9, 48]. The connection between GRP78 retention and increased apoptosis is further strengthened by our findings from CA‐treated PiZ mice that displayed GRP78 retention in AAT globules and higher levels of apoptotic hepatocytes.

Importantly, our study used only male PiZ mice, as they show increased accumulation of inclusions and abundance of mutated protein in the hepatocytes, which is in line with human data since male sex confers PIZZ subjects a higher risk of advanced liver disease [25, 49]. While future studies are needed to fully evaluate the situation in females, GRP78 sequestration is seen in female mice as well (not shown).

Although PiZ transgenic mice are extensively used as a model of AATD‐associated liver disease, differences to the human situation have to be taken into account: Due to multiple insertion of the transgene in PiZ mice, higher amounts of aggregates are found compared to humans [27] who display large interindividual variations in their hepatic AAT content [4]. Although we observed an association of bile acid accumulation with liver injury in PiZ mice as well as in PIZZ subjects, differences in the bile acid composition between mice and humans represent an important limitation, particularly when addressing the importance of specific bile acids [13]. Therefore, studies in animals with a humanised bile acid spectrum are warranted to provide insights into the role of specific bile acids.

In conclusion, our study established several methods of AAT inclusion isolation and demonstrated that GRP78 constitutes a highly abundant component of these aggregates, both in mice and humans. GRP78 retention within the inclusions was promoted by the treatment with CA that mimics the bile acid accumulation occurring in AATD (Figure S10). Notably, increased CA serum levels were seen in subjects with advanced liver fibrosis [50] and we saw increased serum bile acids in subjects with AATD‐associated liver disease. Therefore, increased bile acids may lead to a vicious cycle and a more rapid liver decompensation that occurs in subjects with advanced liver fibrosis and AATD [8].

## Author Contributions

P.S., I.S. and N.G. conceived and designed the study. I.S., N.G., V.U., F.S., S.W.M.O.D., M.B., A.L., L.F, F.M., G.K.E., F.H., M.F. and C.P. contributed to the acquisition of data. I.S., N.G., M.B. and C.P. analysed the data. I.S., N.G., M.B., C.P. and P.S. interpreted the data. I.S., N.G. and P.S. drafted, reviewed and edited the manuscript. Further authors substantially contributed to the final version of the manuscript: V.U., F.S., S.W.M.O.D., M.B., A.L., L.F., F.M., G.K.E., F.N., M.F. and C.P. All authors read and approved the final version of the manuscript.

## Conflicts of Interest

P.S. reports receiving grants and honoraria from Arrowhead Pharmaceuticals, CSL Behring, Grifols Inc., consulting fees or honoraria from Alnylam Pharmaceuticals, Arrowhead Pharmaceuticals, BioMarin Pharmaceutical, BridgeBio, Dicerna Pharmaceuticals, GSK, Intellia Pharmaceuticals, Takeda Pharmaceuticals, Novo Nordisk and Ono Pharmaceuticals, participating in leadership or fiduciary roles in Alpha1‐Deutschland, Alpha1 Global and material transfer support for Vertex Pharmaceuticals and Dicerna Pharmaceuticals.

## Supporting information


**Table S1.** Characteristics of donors of liver explant tissues.
**Table S2.** Antibodies.
**Table S3.** Primers.
Data S1.

**Figure S1.** Characterisation of the analysed mice. Serum AST, ALT and AP levels were measured (A) and H&E (B; a–b), PAS‐D (B; c–d) as well as Sirius red stainings (B, e–f) were performed in *n* = 33 nontransgenic mice (WT) and *n* = 26 littermates overexpressing the human PiZ variant of AAT (PiZ). Scale bar B(a–d) = 100 μm; B(e–f) = 500 μm. Boxplots display median ± IQR, and whiskers indicate the range of the values. **p < 0.01. AAT, alpha‐1 antitrypsin; ALT, alanine aminotransferase; AP, alkaline phosphatase; AST, aspartate aminotransferase, H&E, haematoxylin & eosin; PAS‐D, periodic acid–Schiff‐diastase.

**Figure S2.** Visualisation of AAT inclusions in different fractions of PiZ mouse livers. Liver lysates from PiZ transgenic mice and nontransgenic littermates (WT) underwent fractionation into nuclear (nucl.) and nonnuclear (nonnucl.) fraction with subsequent PAS‐D staining. Scale bar = 500 μm. AAT, alpha‐1 antitrypsin; PAS‐D, periodic acid–Schiff‐diastase.

**Figure S3.** Analysis of primary hepatocytes from nontransgenic mice (WT) and littermates overexpressing human PiZ variant of alpha‐1 antitrypsin (PiZ mice). (A) PAS‐D staining of cultured primary hepatocytes visualises inclusions. (B) Soluble and insoluble fractions of hepatocytes from corresponding animals were examined by immunoblotting with antibodies against AAT and GRP78. K8 was used as a control for loading and fractionation. Scale bar = 100 μm. AAT, alpha‐1 antitrypsin; GRP78, 78 kDa glucose‐regulated protein; K8, keratin 8; PAS‐D, periodic acid–Schiff‐diastase.

**Figure S4.** AAT and GRP78 in insoluble fractions and AAT‐MACS pulldowns from PiZ mouse liver lysates. Triton‐X insoluble fractions as well as AAT‐MACS pulldowns were obtained from livers of PiZ transgenic mice as well as nontransgenic littermates (WT). Immunoblotting was performed with antibodies against AAT and GRP78. AAT, alpha‐1 antitrypsin; GRP78, 78 kDa glucose‐regulated protein; MACS, magnetic activated cell separation.

**Figure S5.** AAT and GRP78 in soluble and insoluble fractions from human adult PIMM subjects and an adult/paediatric PIZZ individual. Human liver explants from a PIZZ adult and a PIZZ child and subjects without the PiZ variant (PIMM genotype) were subdivided into insoluble and soluble fractions followed by immunoblotting with antibodies against AAT and GRP78. AAT, alpha‐1 antitrypsin; GRP78, 78 kDa glucose‐regulated protein.

**Figure S6.** Bile acid composition in PIMM and PIZZ adults. A: principal component analysis (PCA) of bile acid composition between PIMM (red) and PIZZ (green) subjects. Circles represent 95% confidence intervals. Inset shows pairwise permutational analysis of variance (PERMANOVA); B: Volcano plot demonstrating bile acids that are enriched in the serum of PIZZ versus PIMM subjects. Mann–Whitney test was used to compare ranks, y‐axis displays ‐log10 of *q*‐ratio corrected for FDR < 5%. **, *p* < 0.01. GUDCA, glycoursodeoxycholic acid; TCA, taurocholic acid; TCDCA, taurochenodeoxycholic acid; TDCA, taurodeoxycholic acid.

**Figure S7.** Bile acid levels in transgenic mice overexpressing the PiZ variant of alpha‐1 antitrypsin (PiZ) and nontransgenic littermates (WT) fed normal (untr) or cholic acid (CA)–supplemented chow. Concentrations of total bile acids were measured in the sera as well as liver homogenates from the indicated subgroups. Results are displayed as mean ± SD.

**Figure S8.** AAT mRNA and serum levels in PiZ mice fed with normal (untr) or cholic acid (CA)–supplemented chow. PiZ AAT (Z‐AAT) mRNA levels were quantified by RT‐qPCR, with L7 ribosomal mRNA as an internal reference (A), while serum AAT levels were measured via nephelometry (B). Results are displayed as mean ± SD. *p < 0.05. AAT, alpha‐1 antitrypsin, CA, cholic acid, RT‐qPCR, quantitative real‐time polymerase chain reaction.

**Figure S9.** Colocalisation of AAT aggregates and GRP78 in liver sections from a PIZZ child and a PIZZ adult. PAS‐D staining combined with GRP78 immunohistochemistry was used to demonstrate colocalisation of GRP78 with the AAT aggregates in liver sections from a PiZZ child and a PiZZ adult. Scale bar = 50 μm. AAT, alpha‐1 antitrypsin; GRP78, 78 kDa glucose‐regulated protein; PAS‐D, periodic acid–Schiff‐diastase.

**Figure S10.** What our study contributes to understanding the effects of ZAAT and GFR78 aggregation. The accumulation of ZAAT in the endoplasmic reticulum (ER) caused by inherited mutations in the SERPINA1 gene induces proteotoxic stress and predisposes to liver injury and fibrosis (4). The sequestration of important proteins in aggregates is known to promote cell death and disease in various neurologic, muscular and other disorders (1). GRP78 is a key molecular chaperone in the ER, facilitating proper protein folding, preventing the aggregation of misfolded proteins and playing a crucial role in the unfolded protein response (UPR) (9). Our study reveals that GRP78 is a major component of ZAAT aggregates. Bile acid accumulation, which occurs in later stages of human AATD, promotes the retention of GRP78 in the aggregates (Created with BioRender.com). AAT, alpha‐1 antitrypsin; ER, endoplasmic reticulum; GRP78, 78 kDa glucose‐regulated protein.

## Data Availability

The data that support the findings of this study are available from the corresponding author upon reasonable request.
