# Supplementary material for: Alpha‐1 Antitrypsin Inclusions Sequester GRP78 in a Bile Acid–Inducible Manner
Source: Liver Int. 2024 Dec 12;45(1):e16207. doi: 10.1111/liv.16207 (PMC11636636; doi:10.1111/liv.16207)
Supplement: Supplementary file 1 — Table S1. Characteristics of donors of liver explant tissues. Table S2. Antibodies. Table S3. Primers. Data S1. [file LIV-45-0-s001.docx]

**SUPPLEMENTARY MATERIAL**

**Supplementary Materials and Methods**

**Human samples**

PIMM subjects had normal alpha-1 antitrypsin (AAT) serum levels (> 110 mg/dL) and no signs of alpha-1 antitrypsin deficiency (AATD) on liver histology. The presence of PiZ and PiS alleles was excluded by genotyping as previously described (25). Serum levels of aminotransferases, alkaline phosphatase (AP), gamma-glutamyl transferase (GGT), bilirubin and AAT were measured in the Clinical Chemistry Department of Aachen University Hospital.

**Mouse experiments**

Genotyping was carried out from ear sample DNA using primers listed in Table S3. Mice were kept under standardized conditions (12 h day/night cycle, 20-24 °C, humidity 50 %) and had free access to water and food. Sample size for the cholic acid model was calculated using power analysis (G*power V. 3.1.9.2) for a-priori determination based on the SD of previous experiments in wild-type mice. 10 to 12 weeks old male mice were used in the study. They were sacrificed by cervical dislocation after inhalational anesthesia with isoflurane (FORENE, AbbVie AG, Ludwigshafen, Germany). Blood was obtained through cardiac puncture. After centrifugation for 10 min at 5000g, serum was collected and parameters were measured as described in the Clinical Chemistry Department of Aachen University Hospital. Livers were removed, cut and fixed in 10% formaldehyde, snap-frozen in liquid nitrogen or immersed into RNAlater stabilization reagent (Ambion, Life Technologies, Darmstadt, Germany).

**Proteomics**

Six biological replicates of both conditions each (wild-type (WT), PiZ; Triton-X insoluble fractions) and 3 replicates of MACS enrichment were separated on 4-20% gradient gels and stained with Coomassie Brilliant Blue. Gel bands were excised and in-gel digested as described (S1). Tryptic peptides extracted from the gel slices were analyzed by mass spectrometry as described elsewhere (S2). Briefly, peptides were desalted with homemade C18 columns and lyophilized. Dried samples were resuspended in 10% formic acid and subjected to liquid chromatography-coupled mass spectrometry analysis. A) insoluble/high salt: Peptides were loaded on a RSLCnano system and initially trapped for 10 min on a precolumn (Acclaim PepMap100, C18, 5 μm, 100 Å, 300-μm inner diameter × 5 mm, Thermo Fischer, Waltham, MS, USA) in Buffer A (0.1% formic acid in water). The samples were then separated on an analytical column (Acclaim PepMap100, C18, 5 μm, 100 Å, 75-μm inner diameter × 25 cm) using a 70-min gradient (0–10 min, 5% buffer B (80% acetonitrile, 0.1% formic acid); 10–45 min, 10–35% buffer B; 45–47 min, 45–99% buffer B; 47–53 min, 99% buffer B; 53–70 min, 5% buffer B) at 300 nl/min. The peptides were analyzed on an Orbitrap Elite system, which was operated in data-dependent mode. Full-scan MS spectra were acquired in the range of m/z 350–1500 in the Orbitrap of the instrument, using 120000 resolution and a maximum of 1E6 ions AGC (automatic gain control). Collision-induced dissociation (CID) fragmentation was performed in the ion trap on the top seven precursors of each full scan using a collision energy of 35% with a 20 s dynamic exclusion range. B) MACS enriched samples: Peptides were loaded and trapped as above, then separated on a Aurora Elite analytical column (15cm; IonOpticks, Australia) using a 70-min gradient (0–5 min, 2% buffer B (80% acetonitrile, 0.1% formic acid); 5–42 min, 2–35% buffer B; 42-50 min, 35-50% buffer B; 50–53 min, 50–99% buffer B; 53–58 min, 99% buffer B; 58–60 min, 99-2% buffer B, 60–70 min, 2% buffer B) at 250 nl/min. The peptides were analyzed on an Exploris 480 system, which was operated in data-dependent mode with dynamic exclusion set to auto. Full-scan MS spectra were acquired in the range of m/z 350–1250 in the instrument, using 60000 resolution the AGC (automatic gain control) target set to standard. Peptide fragmentation was performed at 15000 resolution on the top 20 precursors of each full scan using a HCD collision energy of 35%. The normalized AGC target was set to 100%.

The raw data files were analyzed via MaxQuant (version 2.0.1.0 (S3)) using the label-free quantification (LFQ) algorithm and the intensity-based absolute quantification of proteins (iBAQ) value. The spectra were searched against the mouse UniProt database version 12/2017, the human entry for AAT (UniProt ID: P01009) containing the E342K mutant was added manually. The search was done using the Andromeda search engine in MaxQuant using default mass tolerance settings. Two missed cleavages were allowed for Trypsin (set as the protease). Carbamidomethylation (Cys) was chosen as fixed and oxidation (Met) as well as and N-terminal protein acetylation as variable modifications. False discovery rate was set to 0.01 for peptides and proteins. Minimum peptide length was seven amino acids. The proteinGroups.txt file derived from MaxQuant was then analyzed and filtered using Perseus (version 1.6.14.0 (S4)). All protein entries corresponding to “reverse” and “only identified by site” were removed. A protein needed to be identified with at least two unique peptides to be further evaluated. The list was filtered to only contain proteins that were identified and quantified in all six samples of at least one of the two genotypes (insoluble/high salt; filtering on LFQ intensity value > 0) or in all 3 samples (MACS enrichment; filtering on iBAQ value > 0). Value imputation based on normal distribution was performed after the last filtering step using the default values in Perseus.

Proteomic analysis of hepatocytes with vs. without inclusions was performed by Oncoprot Platform (TBM-Core US 005, 33000 Bordeaux, France). The liver explant originated from a 10-month-old male PIZZ subject with decompensated liver cirrhosis (METAVIR score F4). Briefly, nine square millimeters of hepatocytes with or without inclusion previously chosen on H&E and PAS+ stained slides were microdissected from a FFPE 10 μm thickness section with a PALM type 4 (Zeiss) automated laser microdissector. Sample preparation and protein digestion were performed as previously described (S5, 26). NanoLC-MS/MS analyses were performed using an ultimate 3000 RSLC Nano-UPHLC system (Thermo Fisher) coupled to a nanospray Q-Exactive hybrid quadrupole-Orbitrap mass spectrometer (Thermo Fisher). Each peptide extract was loaded on a 300 lm IDx5 mm PepMap C 18 precolumn (Thermo Fisher) at a flow rate of 10 l/min. After 3 min desalting, peptides were online separated on a 75 lm IDx25 cm C 18 Acclaim PepMap ® RSLC column (Thermo Fisher) with a 4–40% linear gradient of solvent B (0.1% formic acid in 80% acetonitrile) in 108 min. The separation flow rate was set at 300 nl/min. The mass spectrometer operated in positive ion mode at a 1.8 kV needle voltage. Data were acquired using Xcalibur 3.1 software (Thermo Fisher) in a data-dependent mode. MS scans (m/z 350–1600) were recorded at a resolution of R = 70,000 (@ m/z 200) and an AGC target of 3x10^6^ ions collected within 100 ms. Dynamic exclusion was set to 30s and the top 12 ions were selected from fragmentation in HCD mode. MS/MS scans with a target value of 1x10^5^ ions were collected with a maximum fill time of 100 ms and a resolution of R = 17,500. Additionally, only +2 and +3 charged ions were selected for fragmentation. Other settings were as followed: no sheath and no auxiliary gas flow, heated capillary temperature, 200 C; normalized HCD collision energy of 27% and an isolation width of 2 m/z. Mascot 2.5 algorithm through Proteome Discoverer 1.4 Software (Thermo Fisher) was used in batch mode by searching against the UniProt Homo sapiens database (70,632 entries, Reference Proteome Set, version 07/2016). Two missed enzyme cleavages were allowed. Mass tolerances in MS and MS/MS were set to 10 ppm and 0.02 Da. Oxidation of methionine, acetylation of lysine, and deamidation of asparagine and glutamine were searched as dynamic modifications. Carbamidomethylation on cysteine was searched as static modification. Raw LC-MS/MS data were imported in proline studio 17 for feature detection, alignment, and quantification. Protein identification was accepted only with at least 2 specific peptides with a rank = 1 and with a protein false discovery rate value less than 1% calculated using the ‘decoy’ option in Mascot. Label free quantification of MS1 level by extracted ion chromatograms (XIC) was carried out with parameters indicated previously.

**Fluorescence activated cell sorting (FACS)-based isolation of AAT inclusions**

The isolation of nuclear fractions from mouse liver tissue was performed as mentioned above. The nuclear fraction was dissolved in 1 ml IEB isotonic extraction buffer (IEB) containing protease and phosphatase inhibitors (Roche) and filtered through a 40 µm cell strainer to remove cell debris (Falcon®, Corning Inc., Corning, NY, USA). Inclusions were sorted on a BD FACSAria II SORP Cell Sorter (BD Biosciences, Franklin Lakes, NJ, USA) with the Blue Laser (488 nm). 400V were used for front scatter (FSC) and 350V for side scatter (SSC). The inclusions were isolated based on particle size as well as their granularity by applying a sorting gate of low SSC and a broad range of FSC (20,000-250,000). The sample loading port was set to +4°C and 300 rpm to avoid precipitation. The 70 µm nozzle with 70 psi pressure was used. HBSS (Thermo Fischer) without Calcium and Magnesium was used as sheath fluid for the sorter. The threshold for particle detection was set to 5,000 to exclude cellular debris. The flow rate was set to 22,000 events per second. Sorting of target particles was performed by applying the Precision Mode „Purity“ (Yield Mask 32 / Purity Mask 32 / Phase Mask 0). The collection port was chilled at 4°C and particles were collected in 5 ml glass tubes with 1 ml HBSS. The inclusion-containing solution was centrifuged at 10,000g for 3 hours at +4°C. The pellet was dissolved in 4x reducing Laemmli buffer for subsequent biochemical analysis.

**Magnetic activated cell sorting (MACS)-based isolation of AAT inclusions**

To obtain nuclear fractions, the liver tissue was immersed in a buffer containing 250 mM sucrose, 5 mM Tris-HCl and 5 mM of EDTA (Sucrose-STE-buffer), homogenized and filtered through a 100 µm cell strainer. After centrifugation with 2,000g for 5 min at +4°C, the pellet was washed in Sucrose-STE-buffer and centrifuged thereafter. After resuspension of the pellet in PBS, incubation with an AAT antibody (Sigma A0409) took place at +4°C for 60 min. Buffer consisting of PBS with 2 mM EDTA (PBS-EDTA) was used for washing that was followed by centrifugation for 10 min at 2000g. The step was repeated. The pellet was then resuspended in PBS-EDTA and anti-rabbit IgG magnetic microbeads (Miltenyi Biotec, Bergisch Gladbach, Germany) were added. The preparation of the LS columns was carried out according to the manufacturer’s instructions and the columns were placed on a QuadroMACS™ separator attached to a magnetic MultiStand (Miltenyi). The homogenate carrying the magnetic bead-coupled particles was applied onto the columns. The columns were washed with buffer consisting of PBS-EDTA and 0.5 % Triton-X and flow-through was collected. Finally, the columns were removed from the separator and the isolate was collected by flushing with PBS-EDTA. After centrifugation at 2,000g for 10 min, the isolates were dissolved in 4x reducing Laemmli buffer for subsequent biochemical analyses.

**Quantitative Real-Time Polymerase Chain Reaction (qPCR)**

RNA isolation was performed using the RNeasy mini kit (Qiagen) and complementary DNA was obtained through the reverse transcriptase and oligo-dT method. QPCR was performed with a 7500 fast Real Time PCR Sequence Detection System (Applied Biosystems, Foster City, CA, USA). The primers are listed in Table S3. Ribosomal protein L7 was used as a reference gene and the ddct method was applied for calculation of relative transcript levels.

**Histology**

For histological or immunohistochemical (IHC) staining, liver sections were placed overnight in 4% formaldehyde. After fixation, the samples were dehydrated, embedded in paraffin and cut into 3µm thin sections. Staining was performed with haematoxylin and eosin (H&E) for overall tissue architecture, Sirius red for detection of fibrosis and periodic acid-Schiff-diastase (PAS-D) staining for visualization of AAT aggregates as described previously (S6). IHC was carried out with a mouse ABC Staining System (Vectastain ABC Kit; Vector Laboratories, Burlingame, CA, USA). Slides were deparaffinized, boiled in Antigen Retrieval Solution (Vector) and incubated in 3 % H_2_O_2_ for 10 min for the removal of endogenous peroxidase activity. Subsequent incubations took place in PBS containing 2% bovine serum albumin, the employed antibodies are summarized in Table S2. The incubation with anti-GRP78 antibody was carried out overnight in +4°C. After briefly washing with Tris-buffered saline containing 0.1% Tween-20 (TBST), the slides were incubated with a secondary antibody for 30 min and further washing with TBST was performed. The sections were incubated with Nova Brown peroxidase substrate (Vector) for the development of staining. Images were taken with a light microscope (Leica DM5500B, Solms, Germany) equipped with a digital camera and Leica Application Suite software V4.1 (Leica Microsystems, Heerbrugg, Switzerland).

For fluorescence staining, embedded tissues were cut into 5 µm thick sections. Terminal deoxynucleotidyl transferase–mediated deoxyuridine triphosphate nick-end labeling (TUNEL) of cryosections was performed according to the manufacturer’s instructions (Roche) and nuclei were stained with DAPI (Thermo Fischer). Images were acquired with a fluorescence microscope and the corresponding software (Leica DM5500B).

**Cell culture experiments**

Primary hepatocytes were isolated from 2-3 months old mice as described (S7). The cells were plated on collagen-coated 6-well plates (0,4 x 10^6^ cells / plate) and kept in Hepatozyme-SFM 17705 medium (Thermo Fischer) supplemented with 1% penicillin-streptomycin and L-Glutamine (Thermo Fischer) in a humidified incubator at 37°C and 5% CO_2_. For biochemical analysis, the cells were scratched in cold PBS, pelleted and lysed prior to SDS-PAGE/immunoblotting as described above. Cells from a bronchial epithelial line (IB3) stably expressing non-tagged wild-type (WT) and Z-variant AAT were generated as previously described (S8) and cultured in LHC-8 medium containing 200 µg/ml G418. Cell lysates were prepared in 50 mM Tris-HCl, 150 mM NaCl and 1% Triton X with protease inhibitors (Roche) and protein concentrations were determined by Bradford protein assay (Thermo Fisher). The extract was then resuspended in 1x SDS sample buffer containing dithiothreitol (DTT) and incubated at 95°C for 5 min. For each sample 10-25 mg of total protein was used for SDS-PAGE/immunoblotting as described above.

**Bile acid measurement**

In the serum of human PIMM and PIZZ subjects bile acid levels were determined using LC-MS as described previously (S9) and the total levels were determined as their sum. In brief, serum samples were deproteinized and extracted with 2 volumes of methanol and 1 volume of a mix of deuterated internal standards in isopropanol. Following vigorous mixing and centrifugation (15 min., 50,000g, 4°C), supernatant was transferred to sealable micro-insert glass vials. 5 µl of sample was injected onto a reverse phase column (Acquity UPLC BEH Shield RP18 column, Waters) equilibrated with 85% buffer A (95% H2O, 5% acetonitrile containing 10 mmol/L ammonium acetate). Gradient elution to 100% buffer B (acetonitrile) was performed using an UltimateTM 3000 quaternary UPLC pump (Thermo Scientific) at a flow rate of 0.7 ml/min at 50°C. For detection, a Xevo TQ-S mass spectrometer (Waters) and electrospray ionization was employed. Unweighted linear regression was used to calculate sample concentrations from a 12-point standard curve spiked in plasma with a low endogenous bile acid content.

In mice, the extraction of bile acids from 100 mg liver tissue was carried out as formerly described (S10). The levels of serum and liver bile acids in mice were measured using a total bile acid assay kit (Diazyme 042A-K, Diazyme Laboratories, Poway, CA) according to manufacturer’s instructions.

**Supplementary References**

S1. von Kriegsheim A, Preisinger C, Kolch W. Mapping of signaling pathways by functional interaction proteomics. Methods Mol. Biol. 2008;484:177–192.

S2. Zhu X, Dahlmans V, Thali R, Preisinger C, Viollet B, Voncken JW, et al. AMP-activated Protein Kinase Up-regulates Mitogen-activated Protein (MAP) Kinase-interacting Serine/Threonine Kinase 1a-dependent Phosphorylation of Eukaryotic Translation Initiation Factor 4E. J. Biol. Chem. 2016;291:17020–17027.

S3. Tyanova S, Temu T, Cox J. The MaxQuant computational platform for mass spectrometry-based shotgun proteomics. Nat. Protoc. 2016;11:2301–2319.

S4. Tyanova S, Temu T, Sinitcyn P, Carlson A, Hein MY, Geiger T, et al. The Perseus computational platform for comprehensive analysis of (prote)omics data. Nat. Methods. 2016;13:731–740.

S5. Ezzoukhry Z, Henriet E, Cordelières FP, Dupuy J-W, Maître M, Gay N, et al. Combining laser capture microdissection and proteomics reveals an active translation machinery controlling invadosome formation. Nat. Commun. 2018;9:2031.

S6. Guldiken N, Hamesch K, Schuller SM, Aly M, Lindhauer C, Schneider CV, et al. Mild Iron Overload as Seen in Individuals Homozygous for the Alpha-1 Antitrypsin Pi*Z Variant Does Not Promote Liver Fibrogenesis in HFE Knockout Mice. Cells [Internet]. 2019;8. Available from: http://dx.doi.org/10.3390/cells8111415

S7. Grube J, Woitok MM, Mohs A, Erschfeld S, Lynen C, Trautwein C, et al. ACSL4-dependent ferroptosis does not represent a tumor-suppressive mechanism but ACSL4 rather promotes liver cancer progression. Cell Death Dis. 2022;13:1–13.

S8. Bouchecareilh M, Hutt DM, Szajner P, Flotte TR, Balch WE. Histone deacetylase inhibitor (HDACi) suberoylanilide hydroxamic acid (SAHA)-mediated correction of α1-antitrypsin deficiency. J. Biol. Chem. 2012;287:38265–38278.

S9. García-Cañaveras JC, Donato MT, Castell JV, Lahoz A. Targeted profiling of circulating and hepatic bile acids in human, mouse, and rat using a UPLC-MRM-MS-validated method. J. Lipid Res. 2012;53:2231–2241.

S10. Donepudi AC, Ferrell JM, Boehme S, Choi H-S, Chiang JYL. Deficiency of cholesterol 7α-hydroxylase in bile acid synthesis exacerbates alcohol-induced liver injury in mice. Hepatol Commun. 2018;2:99–112.

S11. Janciauskiene, S., Dominaitiene, R., Sternby, N. H., Piitulainen, E., Eriksson, S. Detection of circulating and endothelial cell polymers of Z and wild type α1-antitrypsin by a monoclonal antibody. J. Biol. Chem. 2002;277:26540–26546.

**Supplementary Tables**

**Table S1 - Characteristics of donors of liver explant tissues**

| Genotype | Age (years) | Sex | Etiology of liver disease |
| --- | --- | --- | --- |
| PIMM | 47 | Male | Amyloidosis in familial Mediterranean fever |
| PIMM | 60 | Female | Chronic hepatitis B |
| PIZZ | 68 | Male | Alpha-1 antitrypsin deficiency |
| PIZZ | 58 | Female | Alpha-1 antitrypsin deficiency |
| PIZZ | 64 | Female | Alpha-1 antitrypsin deficiency |
| PIZZ | 15 | Male | Alpha-1 antitrypsin deficiency |
| PIZZ | 3 | Female | Alpha-1 antitrypsin deficiency, neonatal cholestasis |

**Table S2 - Antibodies**

| Name | Supplier | Cat no. |
| --- | --- | --- |
| AAT (D11) | Self-made | Provided by Prof. Dr. Janciauskiene (S11) |
| AAT | Sigma-Aldrich  Immunology Consultants Laboratory | A0409  GA1T-80A |
| GRP78 | Cell Signaling Technology  Abcam | 3177S  ab21685 |
| GRP94 | Cell Signaling Technology  Cell Signaling Technology | 2104  20292 |
| HDAC2 | Cell Signaling Technology | 2540 |
| Calnexin | Merck | AB2301 |
| Calreticulin | Santa Cruz Biotechnology, Dallas, TX, USA | SC-373863 |
| GAPDH | Novus Biologicals, Littleton, CO, USA  Genetex | NB300-221  GTX627408 |
| Keratin K8 | Developmental Studies Hybridoma Bank; Iowa City, IA, USA | Troma I |
| Goat anti-mouse IgG (H+L) (HRP conjugated) | Invitrogen | G21040 |
| Goat anti-rabbit IgG (H+L) (HRP conjugated) | Invitrogen | G21234 |
| Goat anti-rat IgG (H+L) (HRP conjugated) | Invitrogen | A10549 |
| Rabbit anti-goat IgG (H+L) (HRP conjugated) | Invitrogen | R21459 |
| Rabbit anti-guinea pig IgG (H+L) (HRP conjugated) | Invitrogen | 61-4620 |
| Goat anti-rabbit IgG (H+L) (IRDye 860RD conjugated) | Li-cor | 926-68071 |
| Goat anti-rabbit IgG (H+L) (IRDye 800CW conjugated) | Li-cor | 926-32211 |
| Anti-rabbit IgG microbeads | Miltenyi | 130-048-602 |

**Table S3 - Primers**

| Name | Sequence (5’ -> 3’) |
| --- | --- |
| SERPINA1 (AAT; PiZ) (human) | F: GCAGCCTGACTTCTTTGTGC  R: ATCCTAGGGGGCTTGGTGAT |
| Serpina1a (AAT) (mouse) | F: ATCCCCCTTGGCTCCCATTG  R: GTTTGAATTTGGCGAGCCCCT |
| Heat shock protein 5 (Hspa5, GRP78) (human) | F: CCAACTGTTACAATCAAGGTC  R: ACGAGGAGCAGGAGGAAT |
| Heat shock protein 90 kDa beta member 1 (HSP90B1, GRP94) (human) | F: GGGAGAGTCGTGAAGCAGTTGAG  R: CCACCAAAGCACACGGAGATTC |
| Ribosomal protein L7 (mouse) | F: GAAAGGCAAGGAGGAAGCTCATCT  R: AATCTCAGTGCGGTACATCTGCCT |
| 18s ribosomal RNA (human) | F: GTAACCCGTTGAACCCCATT  R: CCATCCAATCGGTAGTAGCG |

**Supplementary Figure Captions**

**Figure S1 - Characterization of the analysed mice.**

Serum AST, ALT and AP levels were measured (A) and H&E (B; a-b), PAS-D (B; c-d) as well as Sirius red stainings (B, e-f) were performed in n=33 non-transgenic mice (WT) and n=26 littermates overexpressing the human PiZ variant of AAT (PiZ). Scale bar B(a-d) = 100 µm; B(e-f) = 500 µm. Boxplots display median ± IQR, and whiskers indicate the range of the values. **, *P* < 0.01.

AAT, alpha-1 antitrypsin; ALT, alanine aminotransferase; AP, alkaline phosphatase; AST, aspartate aminotransferase, H&E, hematoxylin & eosin; PAS-D, periodic acid-Schiff-diastase.

**Figure S2 - Visualization of AAT inclusions in different fractions of PiZ mouse livers**

Liver lysates from PiZ transgenic mice and non-transgenic littermates (WT) underwent fractionation into nuclear (nucl.) and non-nuclear (non-nucl.) fraction with subsequent PAS-D staining. Scale bar = 500 µm.

AAT, alpha-1 antitrypsin; PAS-D, periodic acid-Schiff-diastase.

**Figure S3 - Analysis of primary hepatocytes from non-transgenic mice (WT) and littermates overexpressing human PiZ variant of alpha1-antitrypsin (PiZ mice)**

(A) PAS-D staining of cultured primary hepatocytes visualizes inclusions. (B) Soluble and insoluble fractions of hepatocytes from corresponding animals were examined by immunoblotting with antibodies against AAT and GRP78. K8 was used as a control for loading and fractionation. Scale bar = 100 µm.

AAT, alpha-1 antitrypsin; GRP78, 78 kDa glucose-regulated protein; K8, keratin 8; PAS-D, periodic acid-Schiff-diastase.

**Figure S4 - AAT and GRP78 in insoluble fractions and AAT-MACS pulldowns from PiZ mouse liver lysates**

Triton-X insoluble fractions as well as AAT-MACS pulldowns were obtained from livers of PiZ transgenic mice as well as non-transgenic littermates (WT). Immunoblotting was performed with antibodies against AAT and GRP78.

AAT, alpha-1 antitrypsin; GRP78, 78 kDa glucose-regulated protein; MACS, magnetic activated cell separation.

**Figure S5 – AAT and GRP78 in soluble and insoluble fractions from human adult PIMM subjects and an adult/pediatric PIZZ individual**

Human liver explants from a PIZZ adult and a PIZZ child and subjects without the PiZ variant (PIMM genotype) were subdivided into insoluble and soluble fractions followed by immunoblotting with antibodies against AAT and GRP78.

AAT, alpha-1 antitrypsin; GRP78, 78 kDa glucose-regulated protein.

**Figure S6 – Bile acid composition in PIMM and PIZZ adults**

A: principal component analysis (PCA) of bile acid composition between PIMM (red) and PIZZ (green) subjects, circles represent 95% confidence intervals. Inset shows pairwise permutational analysis of variance (PERMANOVA); B: Volcano plot demonstrating bile acids that are enriched in the serum of PIZZ vs. PIMM subjects. Mann-Whitney test was used to compare ranks, y-axis displays -log10 of q-ratio corrected for FDR <5%. **, *P* < 0.01.

GUDCA, glycoursodeoxycholic acid; TCA, taurocholic acid; TCDCA, taurochenodeoxycholic acid; TDCA, taurodeoxycholic acid.

**Figure S7 - Bile acid levels in transgenic mice overexpressing the PiZ variant of alpha-1 antitrypsin (PiZ) and non-transgenic littermates (WT) fed normal (untr) or cholic-acid (CA) supplemented chow**

Concentrations of total bile acids were measured in the sera as well as liver homogenates from the indicated subgroups. Results are displayed as mean ± SD.

**Figure S8 - AAT mRNA and serum levels in PiZ mice fed with normal (untr) or cholic acid (CA) supplemented chow**

PiZ AAT (Z-AAT) mRNA levels were quantified by RT-qPCR, with L7 ribosomal mRNA as an internal reference (A), while serum AAT levels were measured via nephelometry (B). Results are displayed as mean ± SD. * *P* <0.05.

AAT, alpha-1 antitrypsin, CA, cholic acid, RT-qPCR, quantitative real-time polymerase chain reaction.

**Figure S9 – Co-localization of AAT-aggregates and GRP78 in liver sections from a PIZZ child and a PIZZ adult**

PAS-D staining combined with GRP78 immunohistochemistry was used to demonstrate co-localization of GRP78 with the AAT aggregates in liver sections from a PiZZ child and a PiZZ adult. Scale bar = 50 µm.

AAT, alpha-1 antitrypsin; GRP78, 78 kDa glucose-regulated protein; PAS-D, periodic acid-Schiff-diastase.

**Figure S10 -** What our study contributes to understanding the effects of ZAAT and GFR78 aggregation.

The accumulation of ZAAT in the endoplasmic reticulum (ER) caused by inherited mutations in the SERPINA1 gene induces proteotoxic stress and predisposes to liver injury and fibrosis (4). The sequestration of important proteins in aggregates is known to promote cell death and disease in various neurologic, muscular and other disorders (1). GRP78 is a key molecular chaperone in the ER, facilitating proper protein folding, preventing the aggregation of misfolded proteins and playing a crucial role in the unfolded protein response (UPR) (9). Our study reveals that GRP78 is a major component of ZAAT aggregates. Bile acid accumulation, which occurs in later stages of human AATD, promotes the retention of GRP78 in the aggregates. (Created with BioRender.com)

AAT, alpha-1 antitrypsin; ER, endoplasmic reticulum; GRP78, 78 kDa glucose-regulated protein; UPR, unfolded protein response.
