# Supplementary figures and images for: Alpha‐1 Antitrypsin Inclusions Sequester GRP78 in a Bile Acid–Inducible Manner
Source: Liver Int. 2024 Dec 12;45(1):e16207. doi: 10.1111/liv.16207 (PMC11636636; doi:10.1111/liv.16207)

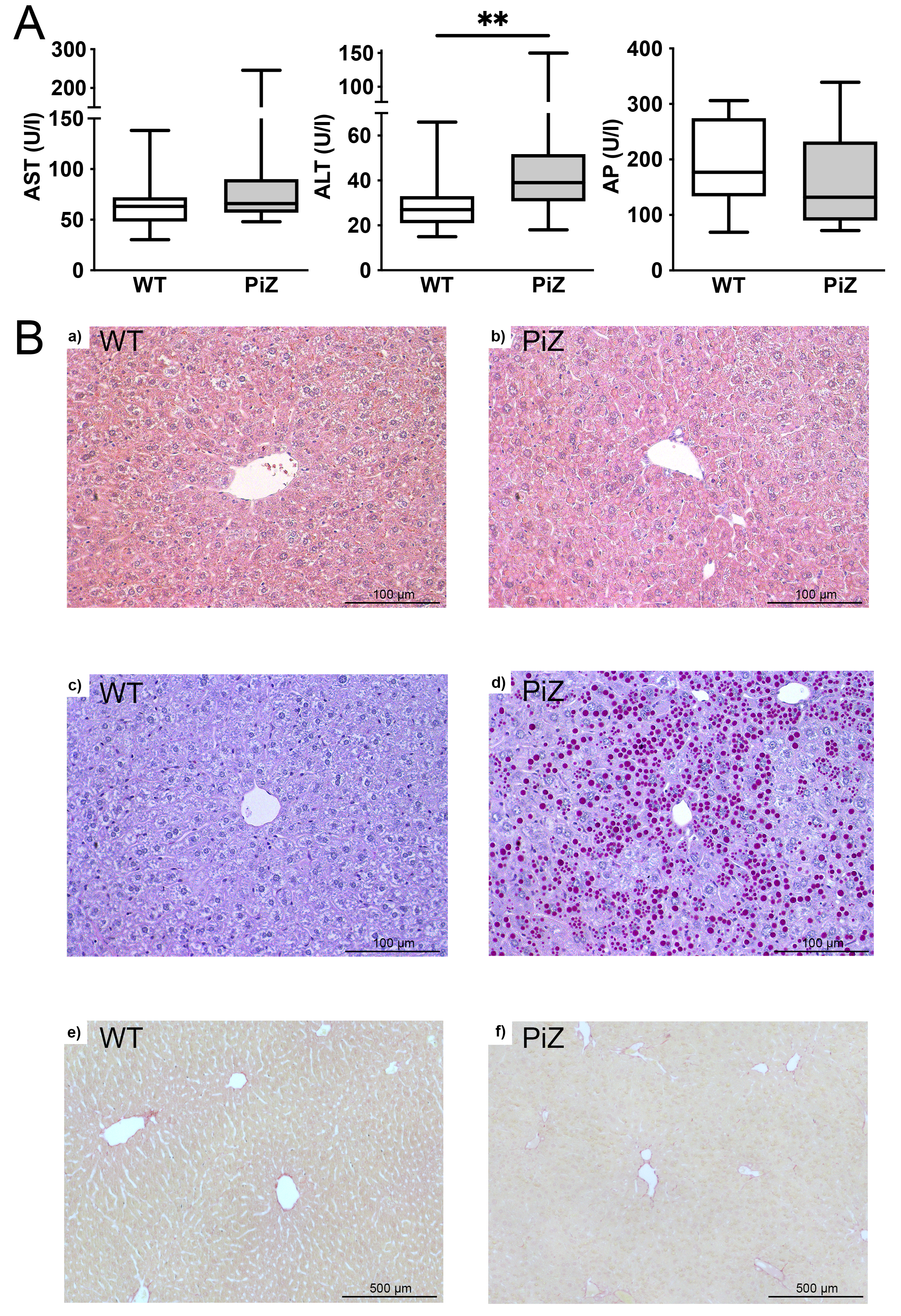

Supplement: Supplementary file 2 — Figure S1. Characterisation of the analysed mice. Serum AST, ALT and AP levels were measured (A) and H&E (B; a–b), PAS‐D (B; c–d) as well as Sirius red stainings (B, e–f) were performed in n = 33 nontransgenic mice (WT) and n = 26 littermates overexpressing the human PiZ variant of AAT (PiZ). Scale bar B(a–d) = 100 μm; B(e–f) = 500 μm. Boxplots display median ± IQR, and whiskers indicate the range of the values. **p < 0.01. AAT, alpha‐1 antitrypsin; ALT, alanine aminotransferase; AP, alkaline phosphatase; AST, aspartate aminotransferase, H&E, haematoxylin & eosin; PAS‐D, periodic acid–Schiff‐diastase. [file LIV-45-0-s010.tif]

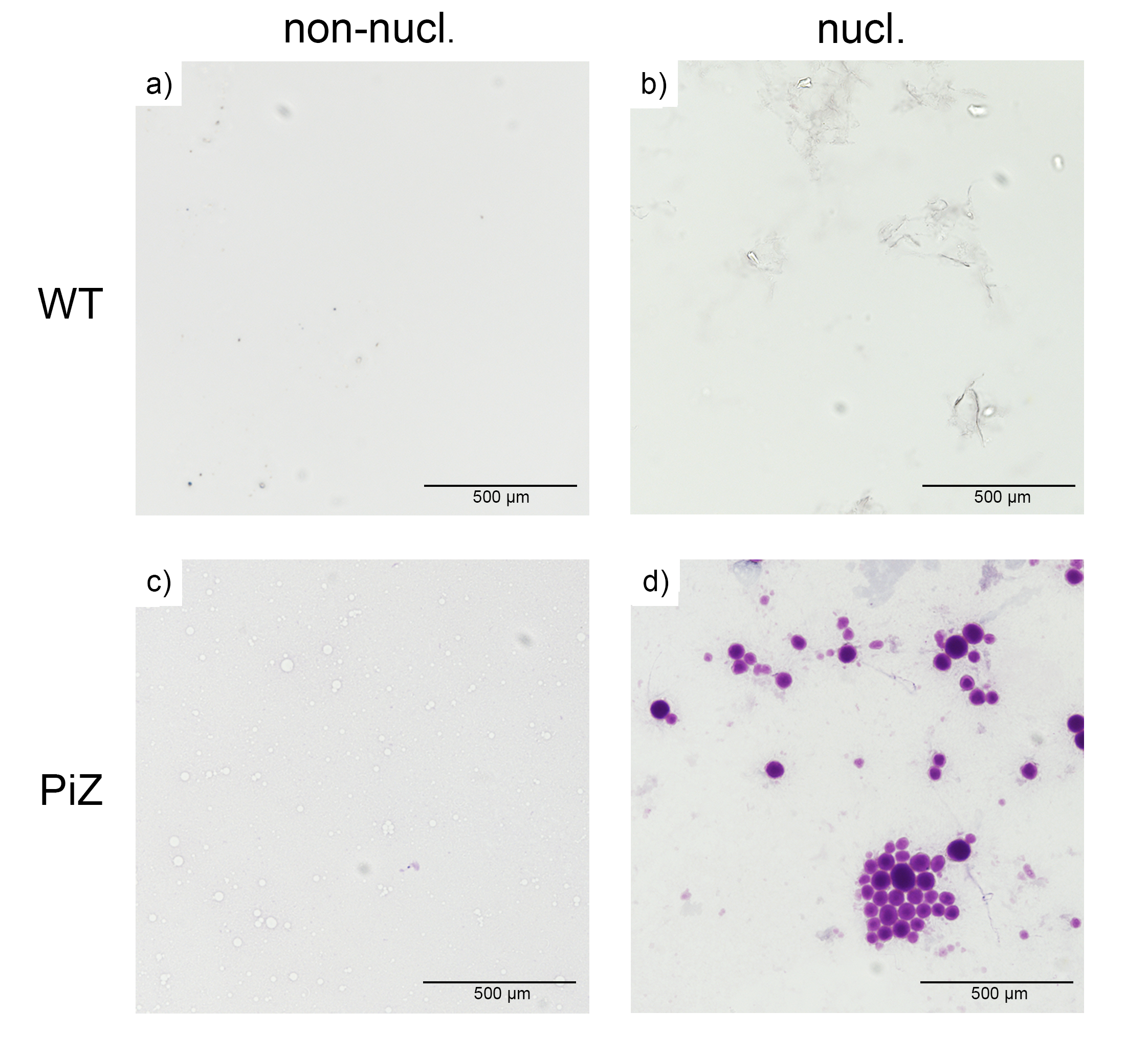

Supplement: Supplementary file 3 — Figure S2. Visualisation of AAT inclusions in different fractions of PiZ mouse livers. Liver lysates from PiZ transgenic mice and nontransgenic littermates (WT) underwent fractionation into nuclear (nucl.) and nonnuclear (nonnucl.) fraction with subsequent PAS‐D staining. Scale bar = 500 μm. AAT, alpha‐1 antitrypsin; PAS‐D, periodic acid–Schiff‐diastase. [file LIV-45-0-s008.tif]

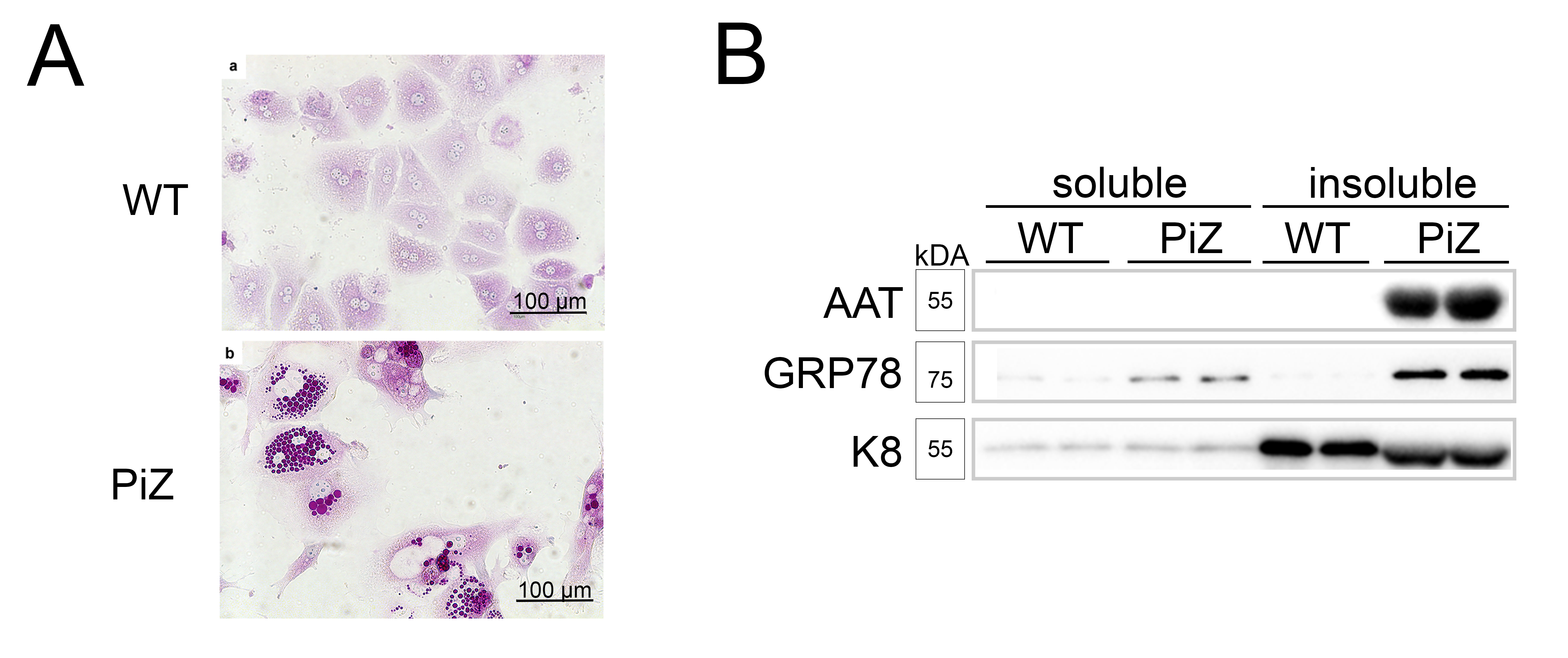

Supplement: Supplementary file 4 — Figure S3. Analysis of primary hepatocytes from nontransgenic mice (WT) and littermates overexpressing human PiZ variant of alpha‐1 antitrypsin (PiZ mice). (A) PAS‐D staining of cultured primary hepatocytes visualises inclusions. (B) Soluble and insoluble fractions of hepatocytes from corresponding animals were examined by immunoblotting with antibodies against AAT and GRP78. K8 was used as a control for loading and fractionation. Scale bar = 100 μm. AAT, alpha‐1 antitrypsin; GRP78, 78 kDa glucose‐regulated protein; K8, keratin 8; PAS‐D, periodic acid–Schiff‐diastase. [file LIV-45-0-s003.tif]

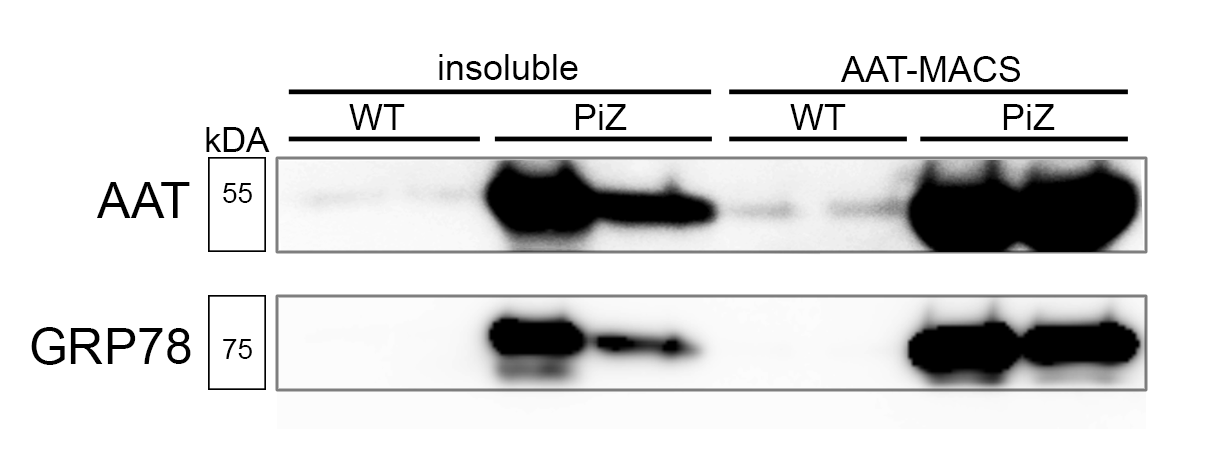

Supplement: Supplementary file 5 — Figure S4. AAT and GRP78 in insoluble fractions and AAT‐MACS pulldowns from PiZ mouse liver lysates. Triton‐X insoluble fractions as well as AAT‐MACS pulldowns were obtained from livers of PiZ transgenic mice as well as nontransgenic littermates (WT). Immunoblotting was performed with antibodies against AAT and GRP78. AAT, alpha‐1 antitrypsin; GRP78, 78 kDa glucose‐regulated protein; MACS, magnetic activated cell separation. [file LIV-45-0-s002.tif]

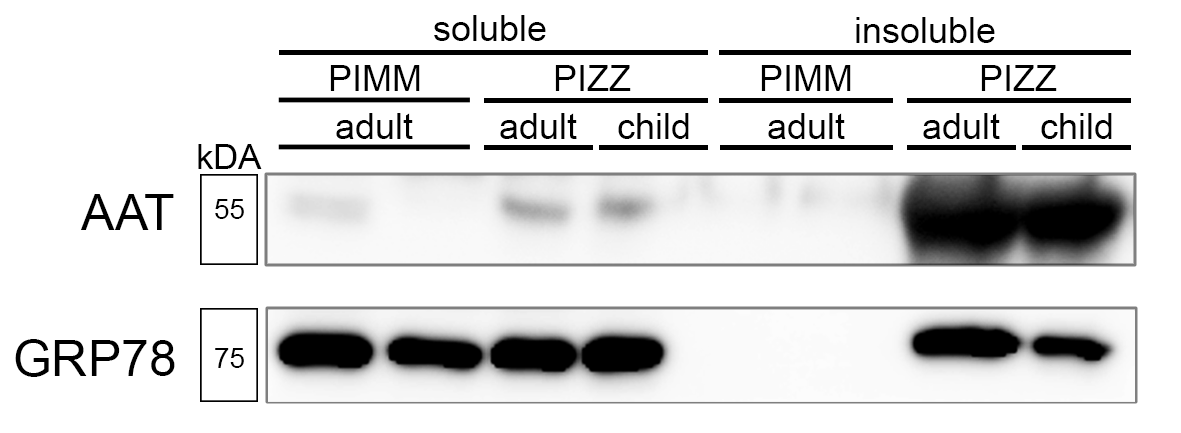

Supplement: Supplementary file 6 — Figure S5. AAT and GRP78 in soluble and insoluble fractions from human adult PIMM subjects and an adult/paediatric PIZZ individual. Human liver explants from a PIZZ adult and a PIZZ child and subjects without the PiZ variant (PIMM genotype) were subdivided into insoluble and soluble fractions followed by immunoblotting with antibodies against AAT and GRP78. AAT, alpha‐1 antitrypsin; GRP78, 78 kDa glucose‐regulated protein. [file LIV-45-0-s007.tif]

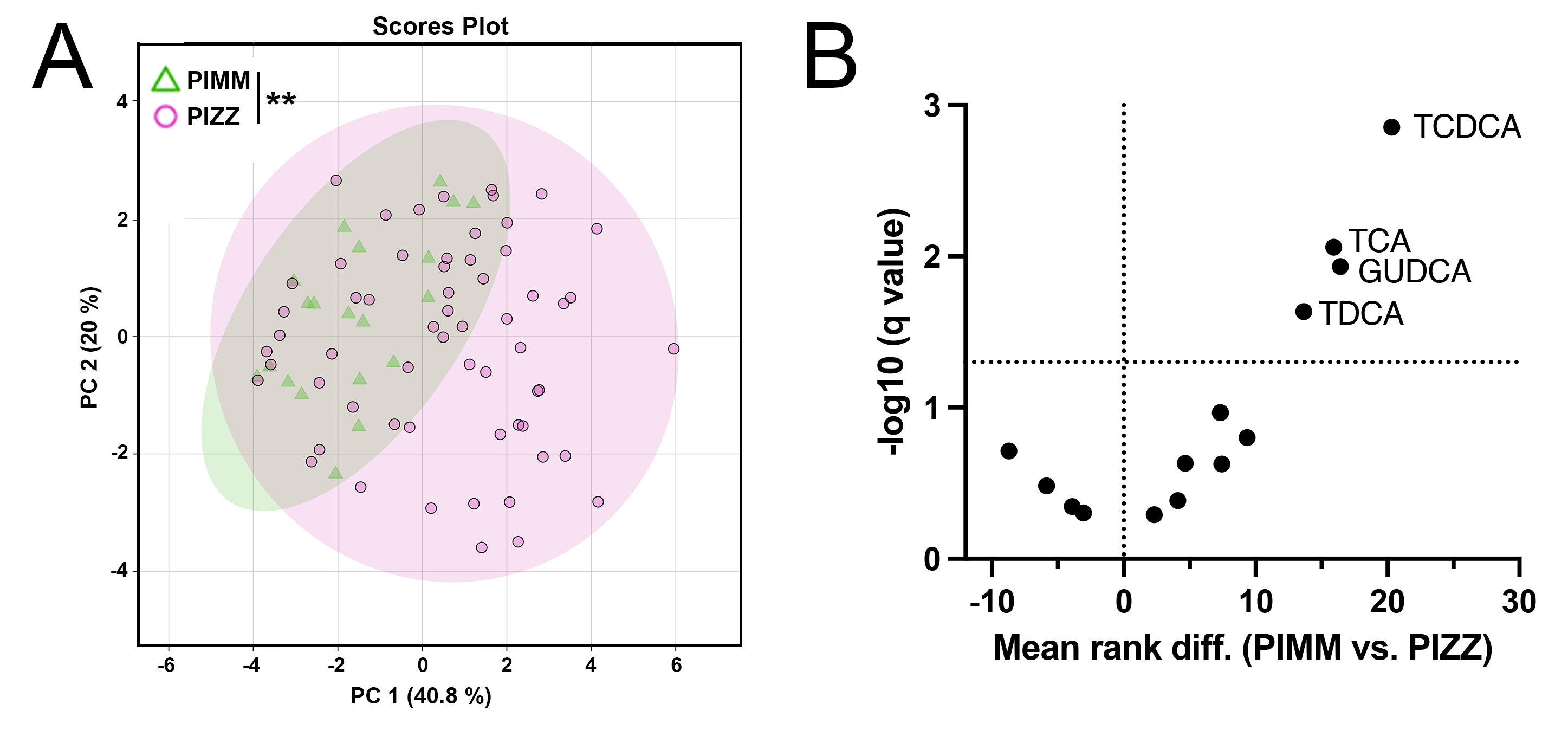

Supplement: Supplementary file 7 — Figure S6. Bile acid composition in PIMM and PIZZ adults. A: principal component analysis (PCA) of bile acid composition between PIMM (red) and PIZZ (green) subjects. Circles represent 95% confidence intervals. Inset shows pairwise permutational analysis of variance (PERMANOVA); B: Volcano plot demonstrating bile acids that are enriched in the serum of PIZZ versus PIMM subjects. Mann–Whitney test was used to compare ranks, y‐axis displays ‐log10 of q‐ratio corrected for FDR < 5%. **, p < 0.01. GUDCA, glycoursodeoxycholic acid; TCA, taurocholic acid; TCDCA, taurochenodeoxycholic acid; TDCA, taurodeoxycholic acid. [file LIV-45-0-s011.tif]

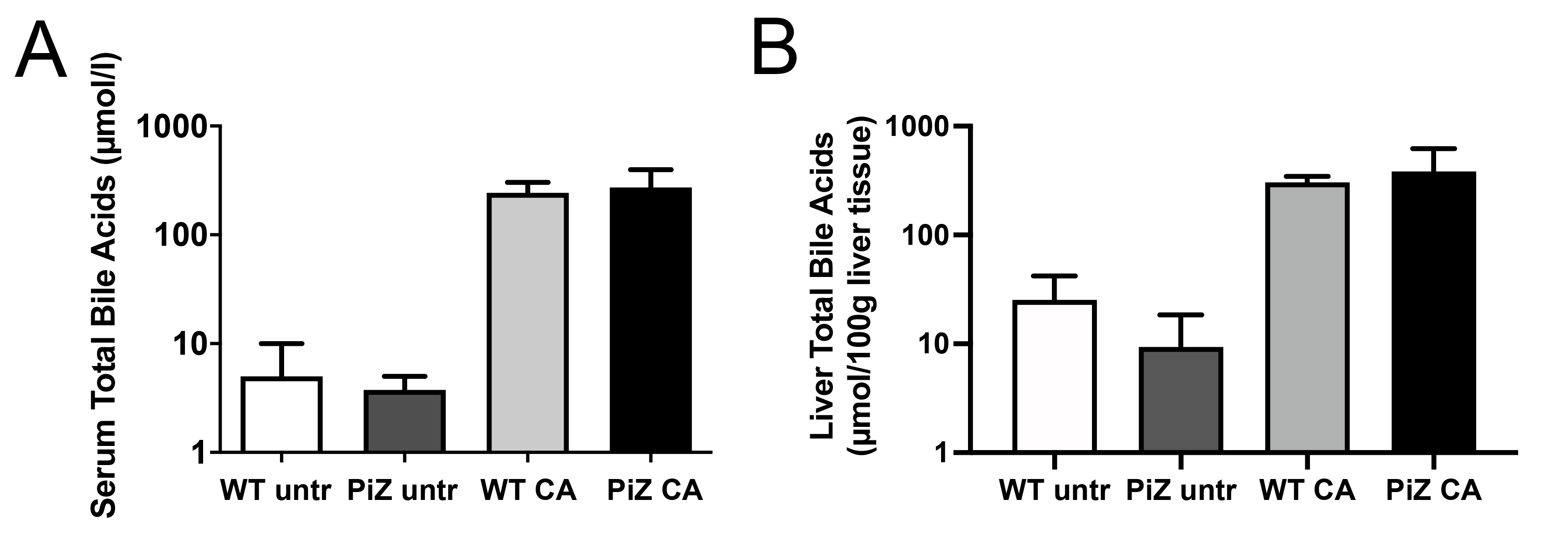

Supplement: Supplementary file 8 — Figure S7. Bile acid levels in transgenic mice overexpressing the PiZ variant of alpha‐1 antitrypsin (PiZ) and nontransgenic littermates (WT) fed normal (untr) or cholic acid (CA)–supplemented chow. Concentrations of total bile acids were measured in the sera as well as liver homogenates from the indicated subgroups. Results are displayed as mean ± SD. [file LIV-45-0-s005.tif]

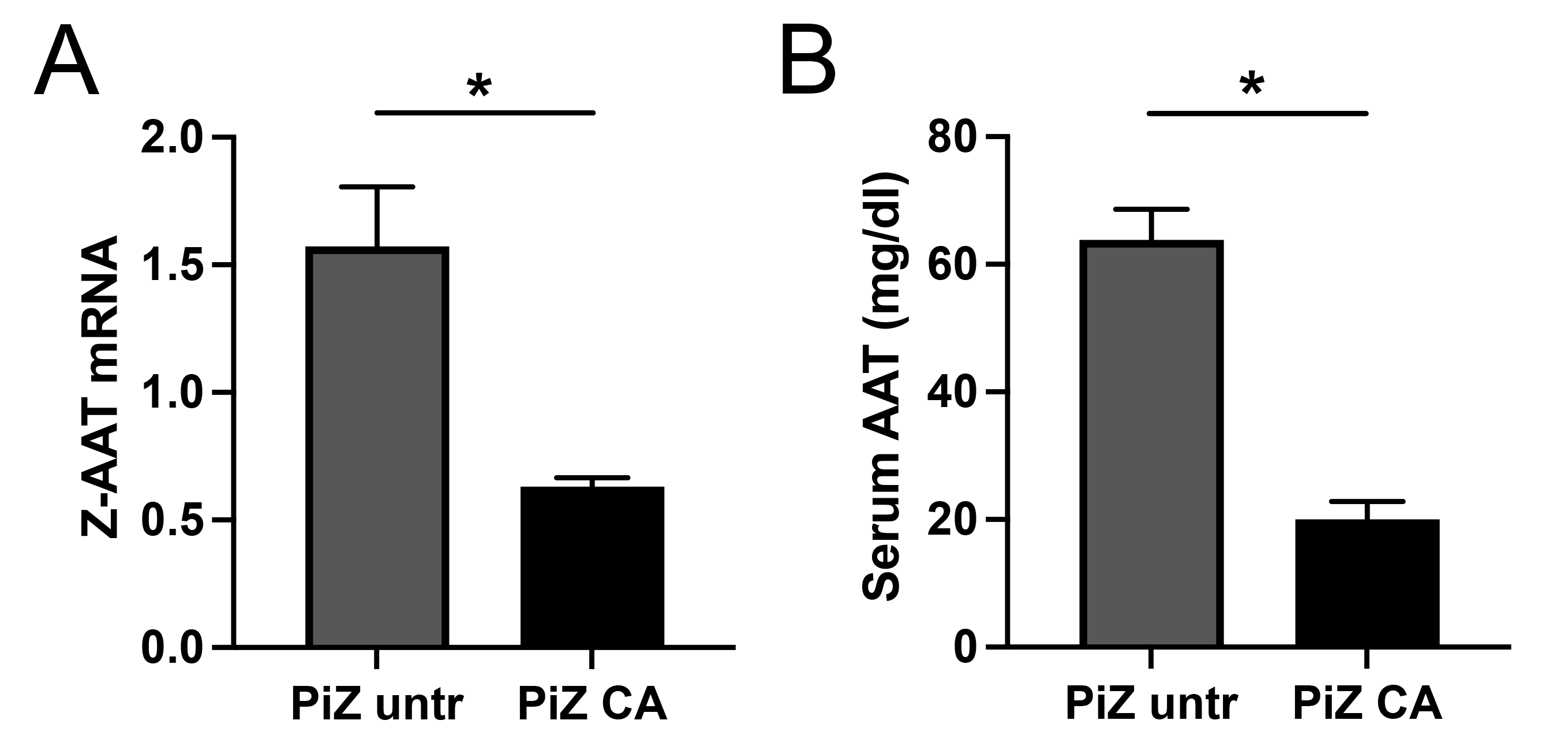

Supplement: Supplementary file 9 — Figure S8. AAT mRNA and serum levels in PiZ mice fed with normal (untr) or cholic acid (CA)–supplemented chow. PiZ AAT (Z‐AAT) mRNA levels were quantified by RT‐qPCR, with L7 ribosomal mRNA as an internal reference (A), while serum AAT levels were measured via nephelometry (B). Results are displayed as mean ± SD. *p < 0.05. AAT, alpha‐1 antitrypsin, CA, cholic acid, RT‐qPCR, quantitative real‐time polymerase chain reaction. [file LIV-45-0-s009.tif]

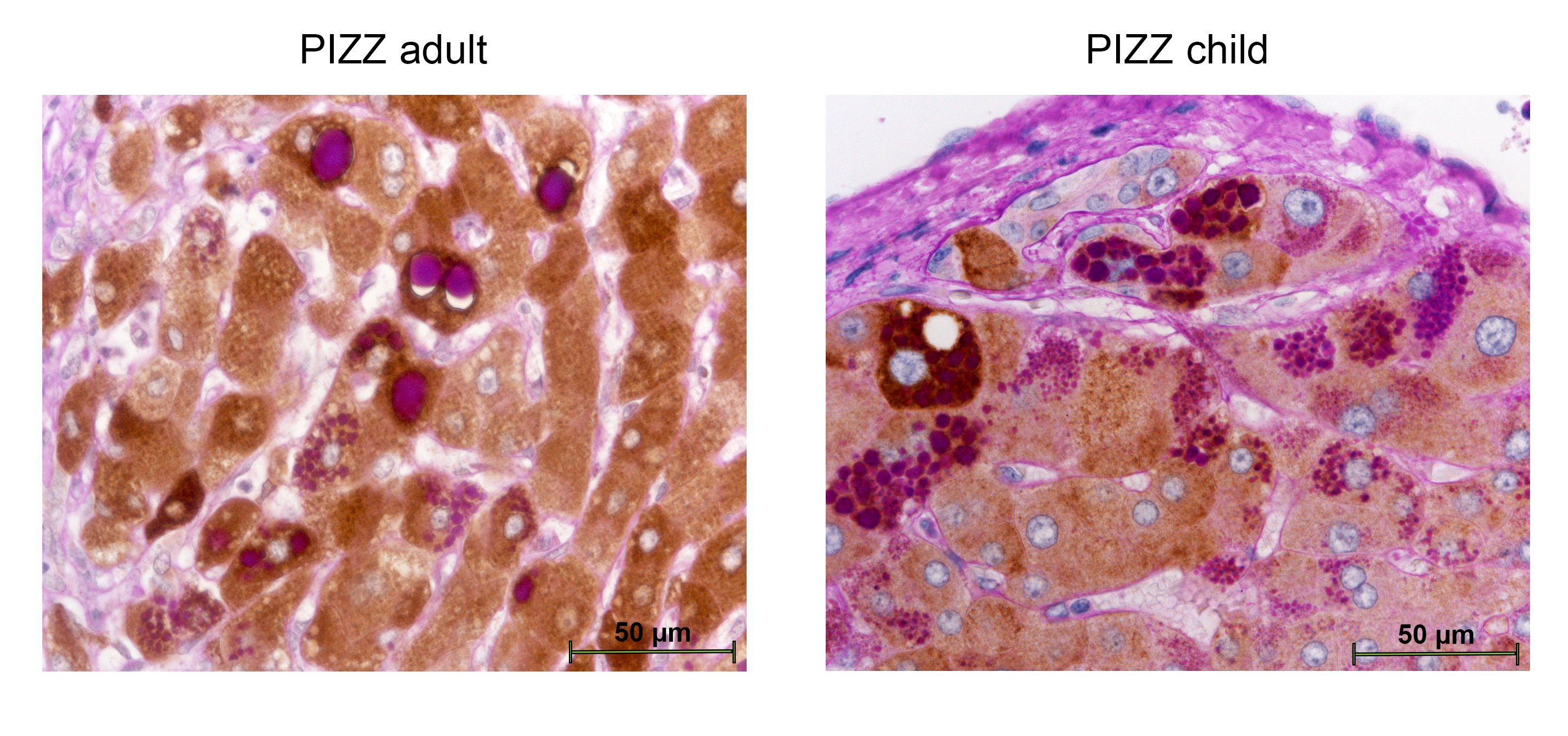

Supplement: Supplementary file 10 — Figure S9. Colocalisation of AAT aggregates and GRP78 in liver sections from a PIZZ child and a PIZZ adult. PAS‐D staining combined with GRP78 immunohistochemistry was used to demonstrate colocalisation of GRP78 with the AAT aggregates in liver sections from a PiZZ child and a PiZZ adult. Scale bar = 50 μm. AAT, alpha‐1 antitrypsin; GRP78, 78 kDa glucose‐regulated protein; PAS‐D, periodic acid–Schiff‐diastase. [file LIV-45-0-s004.tif]

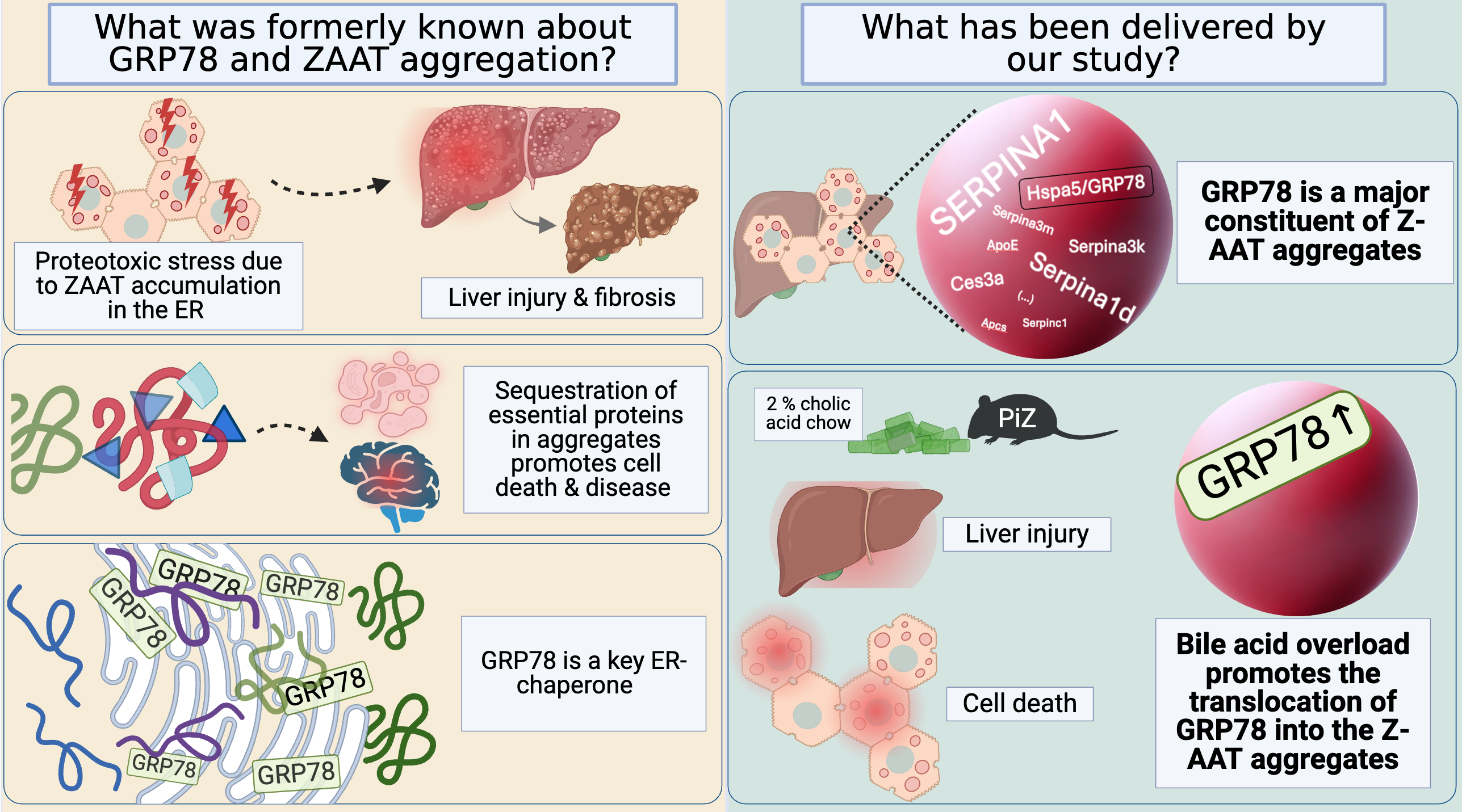

Supplement: Supplementary file 11 — Figure S10. What our study contributes to understanding the effects of ZAAT and GFR78 aggregation. The accumulation of ZAAT in the endoplasmic reticulum (ER) caused by inherited mutations in the SERPINA1 gene induces proteotoxic stress and predisposes to liver injury and fibrosis (4). The sequestration of important proteins in aggregates is known to promote cell death and disease in various neurologic, muscular and other disorders (1). GRP78 is a key molecular chaperone in the ER, facilitating proper protein folding, preventing the aggregation of misfolded proteins and playing a crucial role in the unfolded protein response (UPR) (9). Our study reveals that GRP78 is a major component of ZAAT aggregates. Bile acid accumulation, which occurs in later stages of human AATD, promotes the retention of GRP78 in the aggregates (Created with BioRender.com). AAT, alpha‐1 antitrypsin; ER, endoplasmic reticulum; GRP78, 78 kDa glucose‐regulated protein. [file LIV-45-0-s006.tif]
